# Blood–brain barrier water exchange measurements using contrast‐enhanced ASL

**DOI:** 10.1002/nbm.5009

**Published:** 2023-09-04

**Authors:** Elizabeth Powell, Ben R. Dickie, Yolanda Ohene, Mark Maskery, Geoff J. M. Parker, Laura M. Parkes

**Affiliations:** ^1^ Centre for Medical Image Computing, Department of Medical Physics and Biomedical Engineering University College London London UK; ^2^ Division of Informatics, Imaging and Data Sciences, School of Health Sciences, Faculty of Biology, Medicine and Health University of Manchester Manchester UK; ^3^ Geoffrey Jefferson Brain Research Centre University of Manchester, Manchester Academic Health Science Centre Manchester UK; ^4^ Division of Psychology, Communication and Human Neuroscience, School of Health Sciences, Faculty of Biology, Medicine and Health University of Manchester Manchester UK; ^5^ Department of Neurology Lancashire Teaching Hospitals NHS Foundation Trust Preston UK; ^6^ Queen Square MS Centre, Institute of Neurology University College London London UK; ^7^ Bioxydyn Limited Manchester United Kingdom

**Keywords:** arterial spin labelling, blood–brain barrier, gadolinium‐based contrast agent, permeability, water exchange

## Abstract

A technique for quantifying regional blood–brain barrier (BBB) water exchange rates using contrast‐enhanced arterial spin labelling (CE‐ASL) is presented and evaluated in simulations and in vivo. The two‐compartment ASL model describes the water exchange rate from blood to tissue, $k_{b}$, but to estimate $k_{b}$ in practice it is necessary to separate the intra‐ and extravascular signals. This is challenging in standard ASL data owing to the small difference in $T_{1}$ values. Here, a gadolinium‐based contrast agent is used to increase this $T_{1}$ difference and enable the signal components to be disentangled. The optimal post‐contrast blood $T_{1}$ ( $T_{1,b}^{\text{post}}$) at 3 T was determined in a sensitivity analysis, and the accuracy and precision of the method quantified using Monte Carlo simulations. Proof‐of‐concept data were acquired in six healthy volunteers (five female, age range 24–46 years). The sensitivity analysis identified the optimal $T_{1,b}^{\text{post}}$ at 3 T as 0.8 s. Simulations showed that $k_{b}$ could be estimated in individual cortical regions with a relative error ϵ<1% and coefficient of variation CoV=30%; however, a high dependence on blood $T_{1}$ was also observed. In volunteer data, mean parameter values in grey matter were: arterial transit time $t_{A}=1.15\pm 0.49$ s, cerebral blood flow f=58.0±14.3 mL blood/min/100 mL tissue and water exchange rate $k_{b}=2.32\pm 2.49$ s^−1^. CE‐ASL can provide regional BBB water exchange rate estimates; however, the clinical utility of the technique is dependent on the achievable accuracy of measured $T_{1}$ values.

## Graphical abstract

A technique for quantifying regional blood–brain barrier (BBB) water exchange rates, $k_{b}$, using contrast‐enhanced arterial spin labelling (CE‐ASL) is presented, and evaluated in simulations and in vivo. Simulations quantified the optimally reduced blood $T_{1}$ after contrast, as well as the expected accuracy and precision of $k_{b}$ values measured using CE‐ASL. Proof‐of‐concept data in vivo provided plausible $k_{b}$ estimates; however, values were dependent on the accuracy of measured $T_{1}$ values.

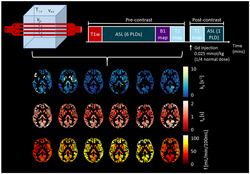

**Abbreviations**
ASL: arterial spin labelling
ATT: arterial transit time
BBB: blood–brain barrier
CASL: continuous arterial spin labelling
CBF: cerebral blood flow
CE‐ASL: contrast‐enhanced arterial spin labelling
CoV: coefficient of variation
CSF: cerebrospinal fluid
DCE: dynamic contrast enhanced
GBCA: gadolinium‐based contrast agent
GM: grey matter
IQR: interquartile range
LD: labelling duration
NEX: number of excitations
PLD: post‐labelling delay
SPGR: spoiled gradient recalled echo
VIF: vascular input function
WM: white matter

## 1 INTRODUCTION

The blood–brain barrier (BBB) plays a vital role in regulating and maintaining healthy brain function. Passive diffusion of solutes and potential neurotoxins from the blood into the brain is tightly restricted, with the transport of necessary metabolites controlled by specialized proteins. Loss of BBB integrity is increasingly indicated in many neurological conditions, including neurodegeneration,^1^, ^2^, ^3^, ^4^ stroke^5^, ^6^ and multiple sclerosis,^7^, ^8^ as well as more generally in ageing.^9^, ^10^, ^11^ Dynamic contrast‐enhanced (DCE) MRI is, at present, the most established MRI method for measuring BBB permeability. When the BBB is damaged, leakage of gadolinium‐based contrast agents (GBCAs) from blood to brain tissue provides a measurable post‐contrast $T_{1}$ enhancement. However, DCE‐MRI is challenging when BBB damage is subtle, as leakage of GBCAs is slow due to the relatively large size of the chelates. Artefacts intrinsic to the method (such as aliasing, signal drift, Gibbs ringing and motion), which can be tolerated when leakage is high, become a limiting factor in detecting the subsequently smaller signal intensity changes for low levels of leakage.^12^

Trans‐BBB water exchange is an alternative MRI‐based biomarker for BBB integrity that has the potential for increased sensitivity to subtle damage.^13^ Several methods have been developed for measuring trans‐BBB water exchange. While some methods have utilized differences in the $T_{1}$ relaxation time^3^, ^4^, ^14^ or intrinsic diffusion properties^15^, ^16^, ^17^ of the blood water directly, many adopt an arterial spin labelling (ASL) approach. Proposed ASL techniques have aimed to separate the intra‐ and extravascular signals using diffusion^18^, ^19^, ^20^ or magnetization transfer^21^ effects, $T_{2}$ properties^11^, ^22^, ^23^, ^24^, ^25^ or velocity encoding.^26^, ^27^ ASL data generally suffer from low SNR, which is addressed by the existing methods in a variety of ways; as such, current techniques are variably limited by long scan times, aggressive smoothing or a lack of regional exchange rate estimates (a comprehensive overview of the different techniques is provided in Reference^13^).

ASL‐based methods utilizing $T_{1}$ differences to separate the intra‐ and extravascular signals are a potential alternative to the above approaches, and preliminary works manipulating the intravascular $T_{1}$ using a GBCA^5^, ^28^, ^29^, ^30^ have shown promise. In the absence of GBCAs, measurements of water exchange using this approach are imprecise owing to the small difference in $T_{1}$ relaxation times between compartments relative to the exchange rate,^31^ requiring SNR levels in excess of clinically attainable values.^32^ ASL data acquired under the influence of an intravascular GBCA benefit from a larger difference between the intra‐ and extravascular $T_{1}$, which should therefore enable the label location to be determined at lower SNR levels. This technique could be a valuable addition to DCE‐MRI studies in cases where minor BBB damage results in minimal GBCA uptake in tissues, providing complementary information on subtle damage via measurements of water exchange.

Contrast‐enhanced (CE) ASL is presented here as a technique for quantifying BBB water exchange, building on previous preliminary data.^29^, ^30^ Simulations are used first to determine the optimal post‐contrast blood $T_{1}$, and then to evaluate the expected accuracy and precision of parameter estimates. Proof of concept is then demonstrated in six healthy volunteers.

## 2 THEORY

**FIGURE 1:**
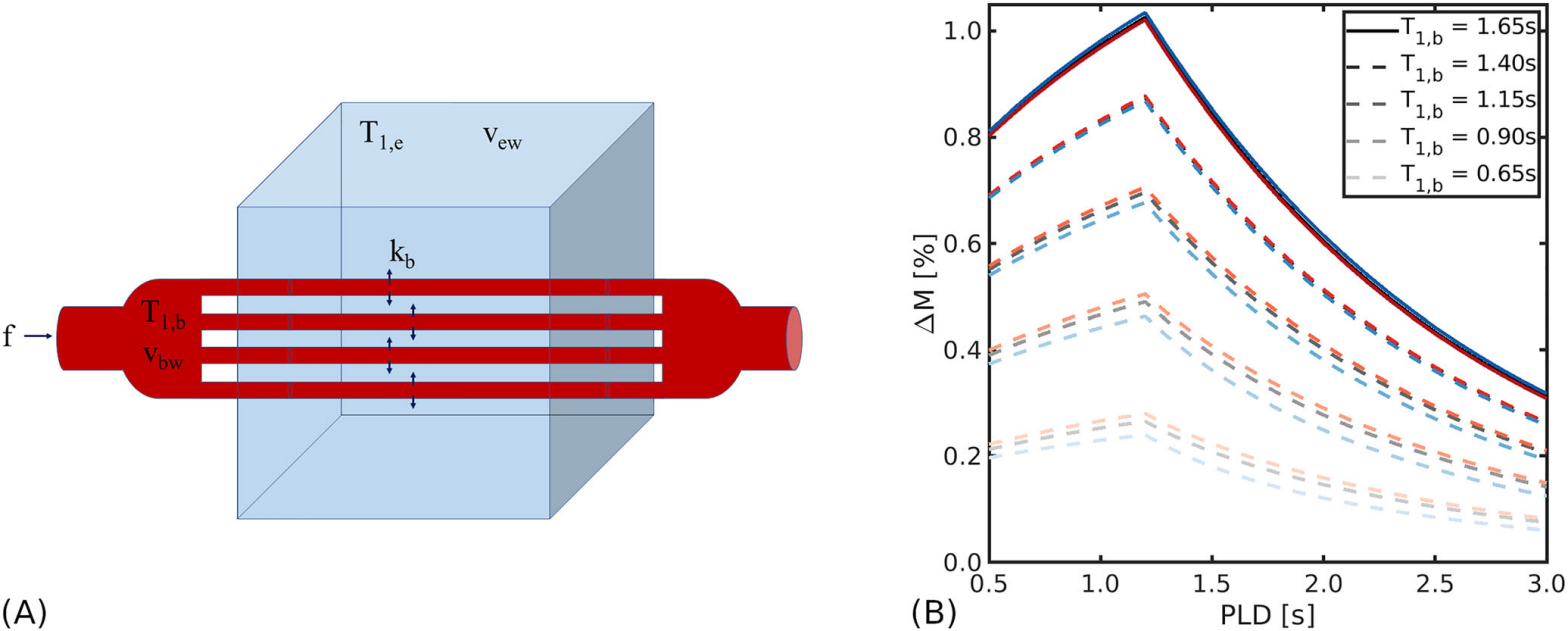
ASL signal model. (A) Schematic diagram of the two‐compartment exchange model. The blood water compartment (red) and extravascular tissue water compartment (blue) have volumes $v_{\text{bw}}$, $v_{\text{ew}}$ and relaxation times $T_{1,b},\;T_{1,e}$ respectively. Exchange occurs at the rate $k_{b}$; cerebral blood flow is indicated by f. (B) Simulated ASL difference signal ΔM for the two‐compartment CASL model in Equation (4) for the equilibrium pre‐contrast $T_{1,b}$ (solid lines) and for a range of post‐contrast $T_{1,b}$ values (dashed lines). Fixed parameters: exchange rate $k_{b}=2.65$ s^−1^ (black/gray lines), extravascular relaxation time $T_{1,e}=1.5$ s, cerebral blood flow f=60 mL blood/ min /100 mL tissue, label duration $t_{L}=2$ s, arterial transit time $t_{A}=1.2$ s, brain:blood partition coefficient λ=0.9 and inversion efficiency α=0.85. The red/blue lines correspond to signals with $k_{b}$ increased/decreased by 50%, respectively (i.e., $k_{b}=3.98,1.33$ s^−1^)

The two‐compartment water exchange model^31^ for continuous arterial spin labelling (CASL) describes the imaging voxel in terms of a blood water compartment and an extravascular tissue water compartment, each with corresponding volumes ( $v_{\text{bw}}$, $v_{\text{ew}}$) and relaxation times ( $T_{1,b},\;T_{1,e}$). Following labelling, tagged blood water arrives at the voxel at arterial transit time (ATT) $t_{A}$, with a cerebral blood flow (CBF) rate f. Labelled water remains in the intravascular compartment for a finite duration before exchanging into the extravascular compartment. Figure 1A shows a schematic diagram of the compartmental model.

Evolution of each compartment's magnetization in the ASL difference image (control − label) is given by

(1) $$\frac{d\left(v_{\text{bw}}\Delta m_{b}\left(t\right)\right)}{dt}=-\frac{v_{\text{bw}}\Delta m_{b}\left(t\right)}{T_{1,b}}+f\Delta m_{a}\left(t\right)-f\Delta m_{v}\left(t\right)+PS\left[\Delta m_{e}\left(t\right)-\Delta m_{b}\left(t\right)\right]$$

(2) $$\frac{d\left(v_{\text{ew}}\Delta m_{e}\left(t\right)\right)}{dt}=-\frac{v_{\text{ew}}\Delta m_{e}\left(t\right)}{T_{1,e}}+PS\left[\Delta m_{b}\left(t\right)-\Delta m_{e}\left(t\right)\right]$$

where PS is the permeability ( P) surface area ( S) product describing exchange between compartments, $\Delta m_{b}$ and $\Delta m_{e}$ represent the magnetization of capillary blood water and extravascular water within the tissue voxel, and $\Delta m_{a}$ and $\Delta m_{v}$ represent the magnetization of arterial blood water and venous blood water arriving at and leaving from the tissue voxel respectively. The total ASL difference signal is modelled as the sum of the intra‐ and extravascular difference magnetizations weighted by their relative volumes:

(3) $$\Delta M\left(t\right)=v_{\text{bw}}\Delta m_{b}\left(t\right)+v_{\text{ew}}\Delta m_{e}\left(t\right).$$

Implicit in Equations (1) and  (2) is the assumption that labelled blood resides in exchanging vessels (capillaries and arterioles) only, meaning that contributions from larger vessels (arteries) are excluded; this is generally expected to be valid for post‐labelling delay (PLD) times greater than 1 s. Further assumptions can be made to simplify the solutions under certain conditions.^31^ First, for perfusion rates in normal human brain tissue, it can be assumed that the label will have decayed (due to $T_{1}$ recovery) before entering the venous circulation, meaning there is no outflow of labelled blood from the voxel during the PLD and so the venous component can be excluded (i.e., $\Delta m_{v}=0$). Second, effects of backflow on the signal—that is, exchange of labelled magnetization from the extravascular space back into the blood—can also be neglected under the assumption that at all times the proportion of labelled extravascular spins is much less than the proportion of labelled intravascular spins (i.e., $\Delta m_{e}\ll \Delta m_{b}$, giving $PS\Delta m_{e}=0$).

Under these assumptions, as derived in earlier work,^31^ the time‐dependent solutions to Equations (1) and  (2) are

(4) $$\Delta M\left(t\right)=\left\{\begin{array}{ll} 0, & t<t_{A}\\ 2fm_{a}^{0}\alpha \exp\left(-R_{1,b}t_{A}\right)\left\{\frac{1-\exp\left(-Jt^{\prime }\right)}{J}\right. & \\ \left.+k_{b}\left[\frac{J-R_{1,e}+R_{1,e}\exp\left(-Jt^{\prime }\right)-J\exp\left(-R_{1,e}t^{\prime }\right)}{JR_{1,e}\left(J-R_{1,e}\right)}\right]\right\}, & t_{A}\leq t\leq t_{A}+t_{L}\\ 2fm_{a}^{0}\alpha \exp\left(-R_{1,b}t_{A}\right)\left\{\left[\frac{1}{J}+\frac{k_{b}}{J\left(R_{1,e}-J\right)}\right]\left[\left(\exp\left(Jt_{L}\right)-1\right)\exp\left(-Jt^{\prime }\right)\right]\right. & \\ \left.-\frac{k_{b}\exp\left(-R_{1,e}t^{\prime }\right)}{R_{1,e}\left(R_{1,e}-J\right)}\left[\exp\left(R_{1,e}t_{L}\right)-1\right]\right\}, & t>t_{A}+t_{L} \end{array}\right.$$

where t is the time from the start of labelling, $t_{L}$ is the labelling duration (LD), $m_{a}^{0}=M_{0}/\lambda$ is the equilibrium arterial magnetization with $M_{0}$ the equilibrium magnetization and λ the brain:blood partition coefficient, α is the inversion efficiency of the labelling, $R_{1,b}=\frac{1}{T_{1,b}}$ and $R_{1,e}=\frac{1}{T_{1,e}}$ are the relaxation rates of the blood and tissue compartments, $J=k_{b}+R_{1,b}$ where the exchange rate of labelled water from blood to tissue is $k_{b}=PS/\upsilon_{\text{bw}}$ and $t^{\prime }=t-t_{A}$. Table 1 gives a full definition of all parameters.

**TABLE 1:** Parameter definitions, abbreviations and units.

Parameter	Definition	Unit
$T_{1,b}^{\text{pre}}$	longitudinal relaxation time of blood pre‐contrast	s
$T_{1,b}^{\text{post}}$	longitudinal relaxation time of blood post‐contrast	s
$T_{1,e}$	longitudinal relaxation time of GM extravascular space	s
f	cerebral blood flow (CBF)	mL blood/min/100 mL tissue
$t_{A}$	arterial transit time (ATT)	s
PS	permeability surface area product to water	mL water/min/mL tissue
$v_{\text{bw}}$	blood water volume fraction	mL water/mL tissue
$v_{\text{ew}}$	extravascular water volume fraction	mL water/mL tissue
$k_{b}$	$k_{b}=PS/\upsilon_{\text{bw}}$	s^−1^
$t_{L}$	label duration (LD)	s
λ	brain:blood partition coefficient	mL/g
α	inversion efficiency of labelling	a.u.
$M_{0}$	equilibrium magnetization	magnetic moment/mL tissue
$m_{a}^{0}$	equilibrium arterial magnetization	magnetic moment/mL blood
$m_{b}$	intravascular magnetization	magnetic moment/mL water
$m_{e}$	extravascular magnetization	magnetic moment/mL water

Longitudinal relaxation during the ATT as the labelled blood water arrives at the imaging slice reduces the magnetization difference $\Delta m_{b}\left(t\right)$ according to Equation (1). Under the influence of an intravascular GBCA, the shorter blood water $T_{1}$ causes $\Delta m_{b}\left(t\right)$ to reduce more rapidly; this allows the presence of magnetization that has permeated into the extravascular space (which now has a substantially longer $T_{1}$ relative to blood) to have a greater influence on the total difference magnetization $\Delta M\left(t\right)$. Figure 1B displays numerical simulations that illustrate the expected $\Delta M\left(t\right)$. With knowledge of the blood and tissue $T_{1}$ before and after GBCA contrast injection, Equations (1) and (2) allow these different $\Delta M\left(t\right)$ to be modelled to extract estimates of the exchange rate. However, as is evident in Figure 1B, higher GBCA concentrations also reduce the overall $\Delta M\left(t\right)$, leading to worsened contrast‐to‐noise ratio. This trade‐off is explored here to identify the optimal conditions for contrast‐enhanced arterial spin labelling (CE‐ASL) estimates of BBB water exchange.

## 3 METHODS

Four simulation experiments were performed to assess the feasibility of the CE‐ASL method and inform acquisition parameters in vivo. The sensitivity of the CE‐ASL signal to water exchange was first evaluated to identify optimal post‐contrast blood water $T_{1}$ ( $T_{1,b}^{\text{post}}$) and PLD times at 3 T. Under these optimal conditions, the impact of inaccurate relaxation time values on parameter estimates was explored in an error analysis. Monte Carlo simulations under varying noise conditions then provided an estimate of the expected accuracy and precision of fitted parameters. Finally, the GBCA dose and time after injection required to obtain optimal $T_{1,b}^{\text{post}}$ values in vivo were calculated using data from a previous study. Based on the simulation results, an in vivo protocol was designed and conducted in six healthy volunteers. All simulations were performed in MATLAB R2019b (The MathWorks Inc., Natick, Massachusetts).

### 3.1 Sensitivity analysis

The sensitivity functions were defined as the partial derivatives of the signal model in Equation (4) with respect to $k_{b}$ (provided in full in Appendix A).

To determine optimal $T_{1,b}^{\text{post}}$ and PLD times, the sensitivity functions were computed for parameter combinations in the ranges $0.15\text{s}\leq T_{1,b}^{\text{post}}\leq 1.65\;\text{s}$  and 0.5s≤PLD≤3.0s , with $T_{1,b}=1.65$ s taken as the non‐contrast‐enhanced value in blood at 3 T.^33^ The exchange rate was fixed at $k_{b}=2.65$ s^−1^, the mean of several published studies.^13^ Calculated sensitivities were normalized using the maximum value obtained across the range of parameter combinations; optimal $T_{1,b}^{\text{post}}$ and PLD times were taken as those that maximized the sensitivity functions.

As the modelling approach assumes that the GBCA remains intravascular, the impact of extravasated contrast agent on sensitivity to $k_{b}$ was assessed by varying $T_{1,e}$ from its non‐contrast‐enhanced value at 1.5 s^34^, ^35^ down to the optimally reduced $T_{1,b}^{\text{post}}$, thereby mimicking GBCA leakage into tissue and the subsequent reduction of $T_{1,e}$. For completeness, this range of $T_{1,e}$ values encompasses the spectrum of exchange rates from no exchange (i.e., $T_{1,e}=1.5$ s) to infinite exchange (i.e., $T_{1,e}=T_{1,b}^{\text{post}}$ s); however, only small reductions are expected for subtle BBB damage. The $T_{1,b}^{\text{post}}$ and PLD times were fixed to their optimized values.

Finally, variation of the sensitivity in relation to underlying exchange rate was explored for $0.5\leq k_{b}\leq 4.0$ s^−1^, which is representative of previously reported values in human grey matter (GM),^13^ over the range $0.15\leq T_{1,b}^{\text{post}}\leq 1.65$ s.

All other model parameters used in each simulation are provided in Table 2.

**TABLE 2:** For all simulations, other fixed parameters were cerebral blood flow f=60 mL blood/min/100 mL tissue,^32^ label duration $t_{L}=2$ s, arterial transit time $t_{A}=1.2$ s^33^ (note that this is variable in vivo depending on labelling location and brain region), brain:blood partition coefficient λ=0.9 ^33^ and inversion efficiency α=0.85.^33^

	$T_{1,b}^{\text{pre}}$ [s]	$T_{1,b}^{\text{post}}$ [s]	$T_{1,e}$ [s]	$k_{b}$ [s^−1^]	PLD [s]
Sensitivity analysis					
‐ optimal $T_{1,b}^{\text{post}}$ & PLD	—	0.15–1.65	1.5	2.65	0.5–3.0
‐ extravasated Gd ( $T_{1,e}$)	—	0.80	0.8–1.5	2.65	1.5
‐ underlying $k_{b}$	—	0.15–1.65	1.5	0.5–4.0	1.5
Error propagation					
‐ $T_{1,b}^{\text{pre}}$ errors	1.65±15%	0.8	1.5	2.65	pre‐contrast: 0.9,
‐ $T_{1,b}^{\text{post}}$ errors	1.65	0.8±15%	1.5	2.65	1.2, 1.5, 1.8, 2.1;
‐ $T_{1,e}$ errors	1.65	0.8	1.5±15%	2.65	post‐contrast: 1.5
					pre‐contrast: 0.9,
Accuracy and precision	1.65	0.8	1.5	0.5–4.0	1.2, 1.5, 1.8, 2.1;
					post‐contrast: 1.5

### 3.2 Error propagation

Systematic biases in parameter estimates arising from $T_{1}$ measurement errors were evaluated using numerical simulations. Noise‐free synthetic signals were generated using Equation (4) for five PLD times between 0.9 and 2.1 s with $T_{1,b}$ set at the equilibrium (pre‐contrast) value and for one PLD of 1.5 s with $T_{1,b}$ set at the optimally reduced (post‐contrast) value. Table 2 provides all model parameters. The signal model was then fitted back to the data using perturbations from ground truth $T_{1}$ values of ±15%. Fitting was performed using least‐squares minimization with f, $t_{A}$ and $k_{b}$ as the free parameters, initialized using 100 starting values and constrained to 0≤f≤200 mL blood/min/100 mL tissue, $0\leq t_{A}\leq 2.5$ s and $0\leq k_{b}\leq 5$ s^−1^. Starting values were randomly distributed between parameter bounds. Resulting errors in f, $t_{A}$ and $k_{b}$ were quantified using the percentage relative error $\epsilon =100\times \left(x_{\text{fit}}-x_{\text{gt}}\right)/x_{\text{gt}}$, where $x_{\text{fit}}$ and $x_{\text{gt}}$ represent the fitted and ground truth value of a given parameter respectively.

### 3.3 Accuracy and precision

The accuracy and precision of fitted parameters were estimated using Monte Carlo simulations under varying noise conditions. Data were simulated before and after contrast (as described for the error propagation) for 25 $k_{b}$ values between 0.5 and 4.0 s^−1^; details of other parameters are in Table 2. For each parameter combination, 2500 control and label signals were synthesized. Zero‐mean Gaussian noise with standard deviations σ=0.0033,0.0017,0.0011 was added to the control and labelled data independently, giving voxel‐wise SNRs of 15, 30, 45 in background‐suppressed control data^36^ (signal taken as 5% of the equilibrium magnetization, assuming 95% background suppression efficiency), before pairwise subtraction to create the difference signal. Corresponding voxel‐wise SNRs in the difference signal were 1.8, 3.6, 5.4. Voxel‐level SNR values were increased by $\sqrt{N}$ to simulate the higher SNR at regional levels, with N=500 taken as the approximate number of voxels in a cortical region of interest (ROI). All $T_{1}$ values were fixed to their ground truth for fitting (performed as for the error propagation). The accuracy of parameter estimates was assessed using the relative error between the ground truth and median fitted values; precision was quantified using the coefficient of variation (CoV), defined as the interquartile range (IQR) of fitted values normalized by the ground truth value. Extreme parameter fits within 5% of the fit constraints ( $0\leq k_{b}\leq 10$ s^−1^) were discarded from these calculations.

To assess the feasibility of $k_{b}$ estimates at different regional levels, voxel‐wise SNR values were adjusted for signal averaging across ROI sizes equivalent to whole lobes ( N=10000) down to the voxel level ( N=1) for a single fixed $k_{b}=2.65$ s^−1^ (fitting constrained to $0\leq k_{b}\leq 5$ s^−1^).

### 3.4 Optimal injected GBCA dose

The GBCA dose needed to achieve the optimal $T_{1,b}^{\text{post}}$ was investigated as a function of injected dose and time post‐injection. Volunteer DCE‐MRI data were taken from a previous study of 31 healthy volunteers (mean age 66 years, range 52–81 years)^37^; briefly, the data comprised pre‐contrast $T_{1}$‐weighted images acquired at three different flip angles, and a dynamic series of single flip angle acquisitions following 0.1 mmol/kg injection of Dotarem GBCA collected every 7.6 s up to 20 min after injection. These data were used to calculate the pre‐ and post‐contrast blood $T_{1}$ values ( $T_{1,b}^{\text{pre}}$ and $T_{1,b}^{\text{post}}\left(t\right)$, respectively); full details can be found in the Supplementary Material of Reference.^37^

The mean vascular input function (VIF)—or blood concentration, $c_{b}\left(t\right)$—over time was calculated using

(5) $$R_{1,b}^{\text{post}}\left(t\right)=R_{1,b}^{\text{pre}}+r_{1}c_{b}\left(t\right),$$

with $R_{1,b}^{\text{post}}\left(t\right)=1/T_{1,b}^{\text{post}}\left(t\right)$ the blood relaxation rate at each time point t after contrast, $R_{1,b}^{\text{pre}}=1/T_{1,b}^{\text{pre}}=0.61$ s^−1^ the blood relaxation rate before contrast and $r_{1}=3.4$ s^−1^mM^−1^ the GBCA longitudinal relaxation coefficient.^38^

The $c_{b}$ estimates were then scaled to 0.25, 0.50 and 0.75 of the value at the full GBCA dose to simulate $R_{1,b}^{\text{post}}$ variation at different dose levels. This provided an estimate of the appropriate dose and time after injection for the optimal $T_{1,b}^{\text{post}}$.

All blood $T_{1,b}^{\text{post}}$ curves were extrapolated using the functional form of the VIF—as described in Reference^39^—to 250 min to show the full $T_{1}$ recovery to its equilibrium (pre‐contrast) value.

### 3.5 MRI acquisition

Proof‐of‐concept data were acquired in six healthy volunteers (five female, mean age 30 years, range 24–46 years) on a simultaneous 3 T SIGNA PET‐MR scanner (GE Healthcare, Chicago, Illinois); ethics approval was granted by the University of Manchester Research Ethics Committee (reference: 2021‐5795‐18124).

A 3D $T_{1}$‐weighted magnetization prepared rapid acquisition gradient echo (MPRAGE) image was acquired prior to contrast agent injection with 1 mm^3^ isotropic resolution for segmentation of GM, white matter (WM) and cerebrospinal fluid (CSF).

**FIGURE 2:**
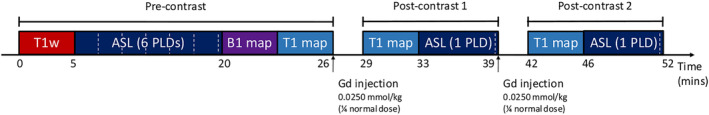
Acquisition pipeline. ASL data (including proton density images acquired with each PLD, indicated by the white dashed lines) and $T_{1}$ maps were acquired pre‐contrast and again following two low‐dose gadolinium injections. A $B_{1}$ map was also collected pre‐contrast.

ASL data and additional $T_{1}$ maps were collected before and after contrast agent injection (Figure 2). Two low‐dose injections of a GBCA (Dotarem) were administered—each a quarter dose (0.025 mmol/kg), providing 0.050 mmol/kg of Dotarem total—in order to capture the optimal $T_{1}$ reduction. Each post‐contrast data set (referred to as PC1 and PC2 respectively) was analysed independently.

ASL was performed with pseudo‐continuous labelling (pCASL), background suppression (all PLDs), no vascular crushing gradients,^33^ a 3D spiral fast spin echo readout with eight spiral interleaves (512 sampling points, giving a spiral readout duration of 475 ms), voxel size 1.7×1.7×4 mm^3^ with 36 axial slices covering the complete brain (lowest slice positioned at the base of the cerebellum), $T_{E}=11$ ms, minimum $T_{R}$ set according to PLD and RF duration/gap of 0.5/1.5 ms. The labelling plane was positioned 2 cm inferior and parallel to the 3D acquisition box. Data at six PLDs (0.7, 0.9, 1.2, 1.5, 1.8 and 2.1 s) were collected before contrast agent injection (the PLD at 0.7 s was not collected in two subjects), with LD 2 s and two repeats (number of excitations, NEX). An additional proton density image was acquired with each PLD. The total pre‐contrast pCASL acquisition time was 15 min. Post‐contrast ASL data were collected approximately 7 min after each contrast agent injection at a single PLD of 1.5 s with NEX=5. One PLD was used to allow time to increase the NEX in comparison to the pre‐contrast acquisition and compensate for the expected signal reduction. The acquisition time of each post‐contrast data set was 6 min.

To produce $T_{1}$ maps before and after contrast, 3D $T_{1}$‐weighted spoiled gradient recalled echo (SPGR) images were acquired using four flip angles (2°, 5°, 15° and 20°), with voxel size 2×2×4 mm^3^, $T_{R}/T_{E}=4.75/1.06$ ms and NEX=8. Acquisition of the four different flip angle images was repeated approximately 3 min after each contrast agent injection, prior to the ASL acquisition. Each flip angle acquisition was 1 min. A 2D Bloch‐Siegert $B_{1}$ map was also collected before contrast with flip angle 10°, field of view matched to the $T_{1}$ map and resolution 3×3×8 mm^3^.

### 3.6 MRI analysis

#### 3.6.1 Extraction of regional ASL and tissue $T_{1}$values

The ASL subtraction images were divided by the ASL proton density images on a voxel‐wise basis. The 3D $T_{1}$‐weighted image was segmented into GM, WM and CSF using SPM12.^40^ Pre‐ and post‐contrast $T_{1}$ maps and the ASL proton density image were co‐registered to the 3D $T_{1}$‐weighted image, and the transformation used to propagate the ASL subtraction images into the same space. The $T_{1}$‐weighted image was then registered to the MNI template, and the transformation applied to the GM and CSF probability maps from the segmentation and the co‐registered $T_{1}$ maps and ASL subtraction images. The automatic anatomical labelling atlas^41^ (masked for GM) was used to extract the mean ASL subtraction signal and $T_{1}$ estimates from the 90 cortical and sub‐cortical regions (excluding the cerebellum).

#### 3.6.2 Estimation of blood $T_{1}$

Pre‐ and post‐contrast $T_{1}$ maps were estimated by fitting the SPGR signal model to the four flip angles at each contrast level.^42^ This was done in R (version 4.2) using the Levenberg–Marquardt optimization solver. Each post‐contrast $T_{1}$ map (in MNI space) was subtracted from the pre‐contrast map to produce two subtraction images, one for each contrast agent dose. On each subtraction image a region in the sagittal sinus and straight sinus was identified using the ROI tool in MRIcro.^43^ The region was masked to contain only those voxels with at least a 20% reduction in $T_{1}$ following contrast agent injection, effectively identifying the voxels with the highest blood volume, to produce the final blood ROI (see Supporting Information, Figure S1). The 75th percentile $T_{1}$ value within the blood ROI on the pre‐contrast $T_{1}$ map was recorded as the $T_{1,b}^{\text{pre}}$ value, chosen as it probably contains high blood volume but will be less affected by noise than the maximum value. The $T_{1,b}^{\text{post}}$ value was estimated by subtracting the pre–post $T_{1}$ difference (again taken as the 75th percentile value within the blood ROI on the subtraction image) from the $T_{1,b}^{\text{pre}}$ value. This process was repeated independently for each subtraction image.

#### 3.6.3 Kinetic modelling of ASL data

Equation (4) was fitted to the data on a voxel‐wise basis in MATLAB 2021a using an unconstrained simplex search method with initial values f=60 mL blood/min/100 mL tissue, $t_{A}=1.0$ s, $k_{b}=1$ s^−1^. Voxel‐wise $T_{1,e}$ values and global $T_{1,b}$ values were fixed to their measured values before and after contrast, with α=0.85, λ=0.9, $t_{L}=2$ s and $M_{0}$ measured from the proton density images. Regional parameter estimates were obtained by taking the median of voxel‐wise values within an ROI.

#### 3.6.4 SNR estimation

No independent noise measurement was available for the in vivo data, so voxel‐level SNR was approximated within each ROI (after averaging signal repetitions) as

(6) $${\text{SNR}}_{\text{vox}}=\frac{\bar{x}}{\sigma }$$

where $\bar{x}$ and σ are the signal mean and standard deviation within an ROI in the ASL difference image at PLD=1.5 s.

## 4 RESULTS

### 4.1 Sensitivity analysis

**FIGURE 3:**
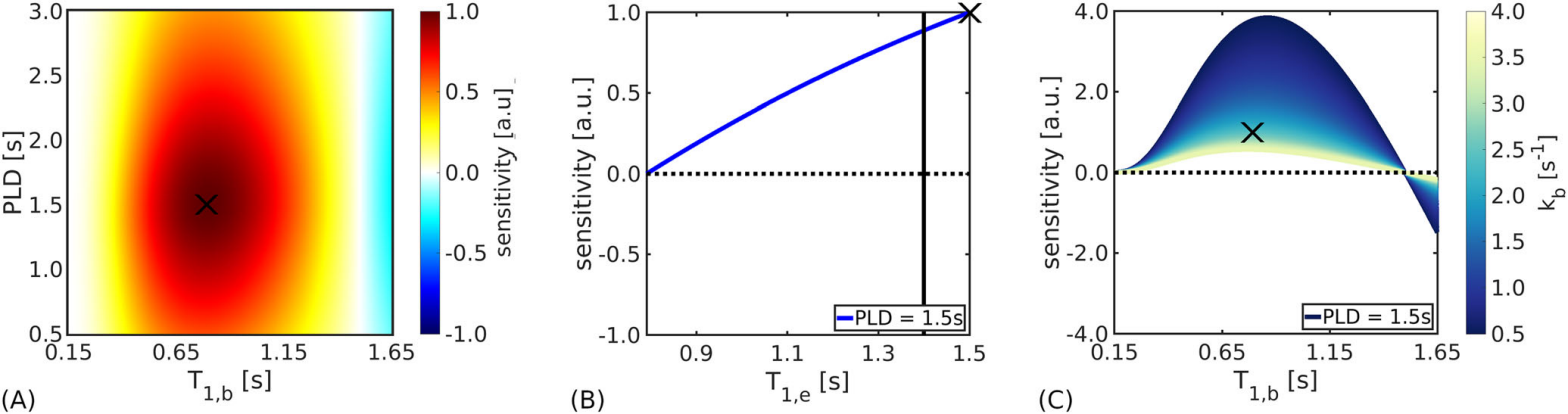
Sensitivity analyses. (A) Sensitivity of the ASL difference signal to the exchange rate, $k_{b}$, as a function of blood $T_{1,b}$ and post label delay (PLD) time (with extravascular $T_{1,e}=1.5$ s and $k_{b}=2.65$ s^−1^). The colour bar shows the magnitude of the sensitivity functions, which were normalized using the maximum value obtained over the range of parameter combinations (indicated by the black cross). (B) Sensitivity dependence on $T_{1,e}$, simulating the effect of extravasated contrast agent (with $T_{1,b}=0.8$ s, PLD=1.5 s, $k_{b}=2.65$ s^−1^). The black line indicates the potential $T_{1,e}$ after leakage from minor blood‐brain barrier damage in vivo. (C) Sensitivity dependence on underlying $k_{b}$ values (with $T_{1,b}=0.8$ s, $T_{1,e}=1.5$ s, PLD=1.5 s). All sensitivity functions were normalized to the parameter set consisting of $k_{b}=2.65$ s^−1^, $T_{1,b}=0.8$ s, $T_{1,e}=1.5$ s and PLD=1.5 s (indicated in each panel by the black cross). Other model parameter details are given in Table 2.

Figure 3A shows that the model was most sensitive to $k_{b}$ for $T_{1,b}=0.8$ s and PLD=1.5 s (normalized sensitivity value of 1). These optimal values provided a more than threefold increase in sensitivity compared with the use of no contrast (equivalent to $T_{1,b}=1.65$ s; normalized sensitivity value of −0.3). Sensitivity remained within 90% of the maximum value over the ranges $0.6\leq T_{1,b}\leq 1.0$ s and 1.3≤PLD≤1.9 s. Minimal sensitivity (under 10% of the maximum value) was observed both for very short $T_{1,b}$ values ( $T_{1,b}\leq 0.3$ s) and for $T_{1,b}\approx T_{1,e}$.

Reductions in $T_{1,e}$ arising from extravasated contrast agent corresponded to an approximately linear decrease in sensitivity, culminating in zero sensitivity to $k_{b}$ for $T_{1,e}=T_{1,b}$ (Figure 3B). This represents the full range of BBB integrity, from fully intact with no leakage of the GBCA (i.e., $T_{1,e}=1.5$ s, the equilibrium value) to unobstructed leakage (i.e., $T_{1,e}=T_{1,b}$). A reduction of ∼0.1 s may be expected for minor BBB damage,^37^ corresponding to a decrease in sensitivity of ∼10%.

Figure 3C shows the sensitivity dependence of the model to underlying $k_{b}$ values. Greater sensitivity was observed for lower exchange rates. From the magnitude of the sensitivity function—which provides an indication of the expected level of measurement precision for a given noise level—it can be seen that, compared with a baseline $k_{b}=2.65$ s^−1^, an increase of 15% in the exchange rate to $k_{b}=2.92$ s^−1^ would correspond to a 12% reduction in measurement precision. The optimal $T_{1,b}$ varied minimally from $T_{1,b}=0.77$ s at the highest exchange rate ( $k_{b}=4.0$ s^−1^) to $T_{1,b}=0.86$ s at the slowest exchange rate ( $k_{b}=0.5$ s^−1^).

### 4.2 Error propagation

**FIGURE 4:**
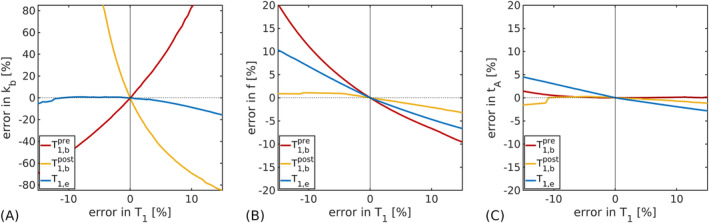
Error propagation. (A) Error propagated into the exchange rate, $k_{b}$, from errors in each measured $T_{1}$ value (blood pre‐contrast, $T_{1,b}^{\text{pre}}$; blood post‐contrast, $T_{1,b}^{\text{post}}$; extravascular, $T_{1,e}$). (B) Error propagated into the cerebral blood flow, f, from errors in each $T_{1}$ value. (C) Error propagated into the arterial transit time, $t_{A}$, from errors in each $T_{1}$ value. Ground truth parameter values (all figures): $k_{b}=2.65$ s^−1^, f=60 mL blood/min/100 mL tissue, $t_{A}=1.2$ s, $T_{1,b}^{\text{pre}}$=1.65 s, $T_{1,b}^{\text{post}}=0.8$ s, $T_{1,e}=1.5$ s (full details in Table 2)

Figure 4 shows the errors propagated into $k_{b}$, f and $t_{A}$ by errors in measured $T_{1}$ values. The accuracy of $k_{b}$ was highly sensitive to errors in both $T_{1,b}^{\text{pre}}$ and $T_{1,b}^{\text{post}}$: to obtain estimates of $k_{b}$ with less than 10% error required $T_{1,b}^{\text{pre}}$ to be known within ±1.5% and $T_{1,b}^{\text{post}}$ within ±0.7%. Errors in $T_{1,e}$ propagated less uncertainty into $k_{b}$ estimates, requiring a measurement accuracy of ±11% to maintain the same 10% error level in $k_{b}$.

CBF accuracy was more influenced by errors in $T_{1,b}^{\text{pre}}$ and $T_{1,e}$ than in $T_{1,b}^{\text{post}}$. To estimate f with under 10% error required a measurement accuracy within ±9.1% for $T_{1,b}^{\text{pre}}$ and within ±14.4% for $T_{1,e}$. The CBF error was under 5% for all simulated $T_{1,b}^{\text{post}}$ errors ( ±15%).

The error propagated into ATT estimates was under 5% for all simulated $T_{1}$ errors ( ±15%).

### 4.3 Accuracy and precision

**FIGURE 5:**
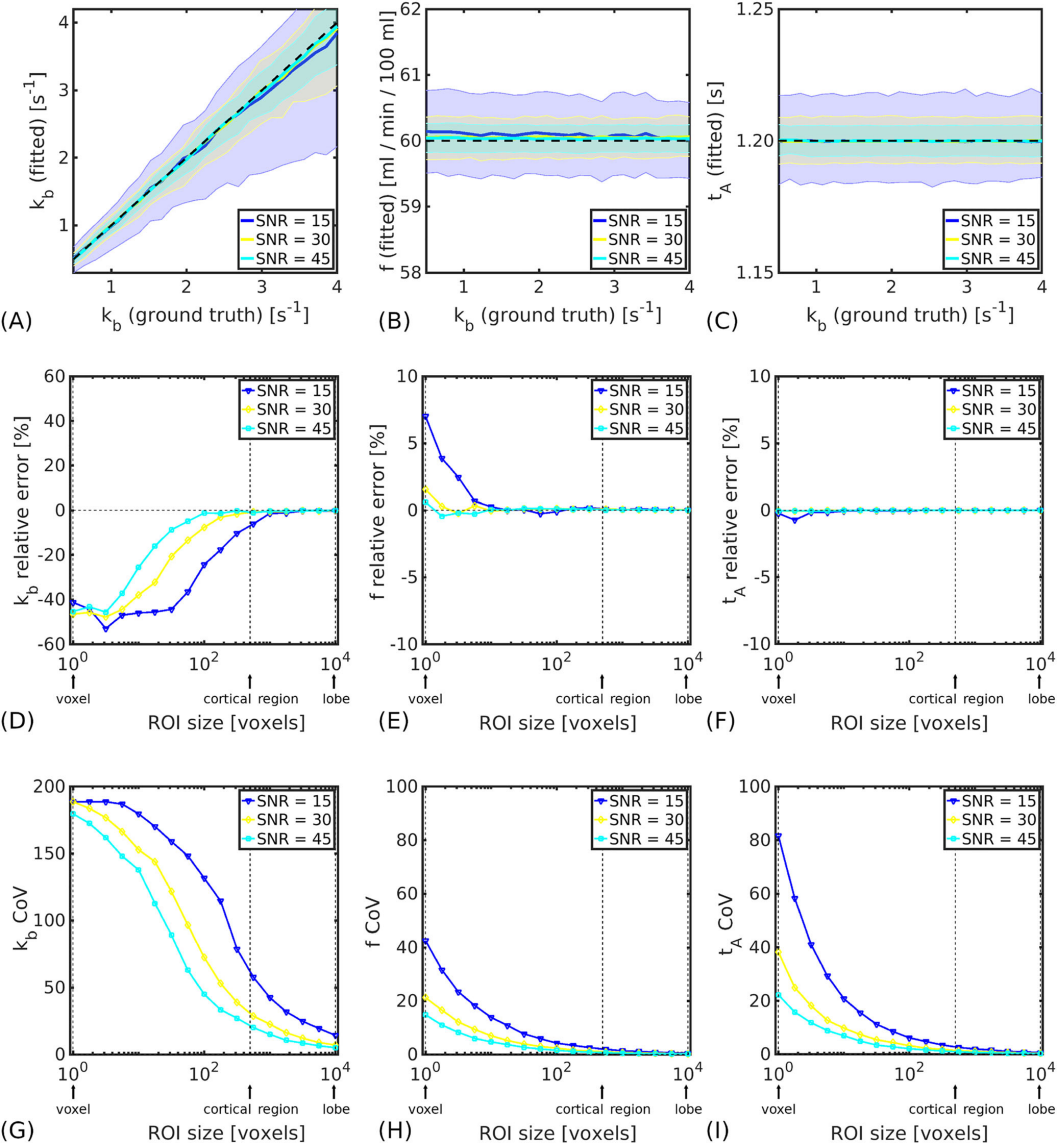
Accuracy and precision. (A–C) Median parameter values (solid lines) for a simulated cortical ROI (500 voxels) as a function of ground truth exchange rates in the range $0.5\leq k_{b}\leq 4.0$ s^−1^ for the fitted exchange rate, $k_{b}$ (A), cerebral blood flow, f (B) and arterial transit time $t_{A}$ (C). Shaded regions indicate the IQR of fitted values; black dashed lines indicate ground truth parameter values. (D–F) Relative errors in parameter estimates (for fixed $k_{b}=2.65$ s^−1^) after signal averaging across different simulated ROI sizes for $k_{b}$ (D), f (E) and $t_{A}$ (F). (G–I) The coefficient of variation (CoV) of parameter estimates (for fixed $k_{b}=2.65$ s^−1^) after signal averaging across different simulated ROI sizes for $k_{b}$ (G), f (H) and $t_{A}$ (I). In all figures, displayed SNR levels indicate voxel‐wise values in the control signal. Full simulation details are in Table 2.

The variation in accuracy and precision of fitted model parameters with underlying $k_{b}$ values is shown in Figure 5A–C. No biases were evident in any of the $k_{b}$ estimates; however, precision, indicated by the shaded error bars (IQR of fitted values), was reduced at higher $k_{b}$ values, as predicted by the sensitivity analysis in Figure 3C. The accuracy and precision of the CBF and ATT were largely unaffected by underlying exchange rates. The number of extreme fits was under 6% in all cases (see Supporting Information, Figure S2).

The feasibility of regional water exchange measurements is considered in Figure 5D–I. Given a voxel‐wise SNR of 30 in the control signal (and fixed $k_{b}=2.65$ s^−1^), in a cortical ROI (500 voxels) the relative error of $k_{b}$ was under 1% and the CoV was 30%. Signal averaging across a simulated lobe (10 000 voxels) reduced the CoV to 7%. The CoV of voxel‐level $k_{b}$ estimates was very high (190%). CBF and ATT were estimated with good accuracy (relative error ϵ<1%) and reasonable precision ( ${\text{CoV}}_{f}<21$% and ${\text{CoV}}_{t_{A}}<38$%) at the voxel level for SNR=30.

### 4.4 Optimal injected GBCA dose

**FIGURE 6:**
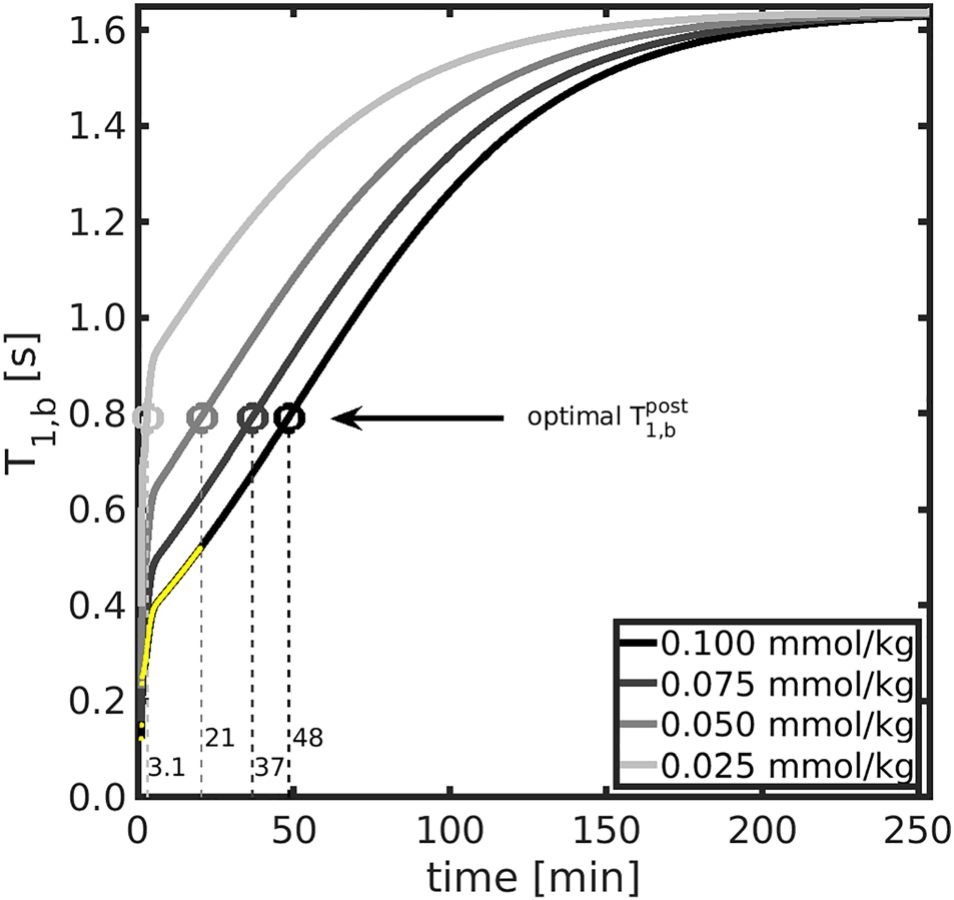
Optimal injected GBCA dose. Calculated blood $T_{1,b}$ recovery curves post‐contrast are shown for different GBCA dose levels; a standard full dose is 0.100 mmol/kg. Dotted lines indicate the time after injection to reach the optimal $T_{1,b}^{\text{post}}=0.8$ s, highlighted for each dose level by the circles. Yellow markers indicate the experimental data from Reference^37^.

Figure 6 shows the recovery of $T_{1,b}^{\text{post}}$ with time after contrast agent injection. Optimal values of $T_{1,b}^{\text{post}}=0.8$ s were obtained approximately 3 min after injection of a 0.025 mmol/kg dose (quarter dose), 21 min after a 0.050 mmol/kg dose (half dose), 37 min after a 0.075 mmol/kg dose (three‐quarter dose) and 48 min after a 0.100 mmol/kg dose (full dose).

### 4.5 In vivo data

The mean and standard deviation across subjects of GM $T_{1}$ values were: (i) $T_{1,e}=1.50\pm 0.09$ s pre‐contrast; (ii) $T_{1,e}=1.48\pm 0.09$ s for PC1, and; (iii) $T_{1,e}=1.39\pm 0.09$ s for PC2. Representative tissue $T_{1}$ maps pre‐ and post‐contrast, along with example ASL subtraction images pre‐ and post‐contrast, are provided in the Supporting Information, Figure S3. Pre‐ and post‐contrast blood $T_{1,b}$ values were: (i) $T_{1,b}^{\text{pre}}$=1.87 ±0.06 s and $T_{1,b}^{\text{post}}=1.14\pm 0.11$ s for PC1, and; (ii) $T_{1,b}^{\text{pre}}$=1.83 ±0.06 s and $T_{1,b}^{\text{post}}=0.91\pm 0.04$ s for PC2.

As the PC2 $T_{1,b}^{\text{post}}$ best approximated the optimal value, results from the PC2 data set will primarily be presented from here on; results from the PC1 data can be found in the Supporting Information (Figure S4.1 and Table S4.2).

**FIGURE 7:**
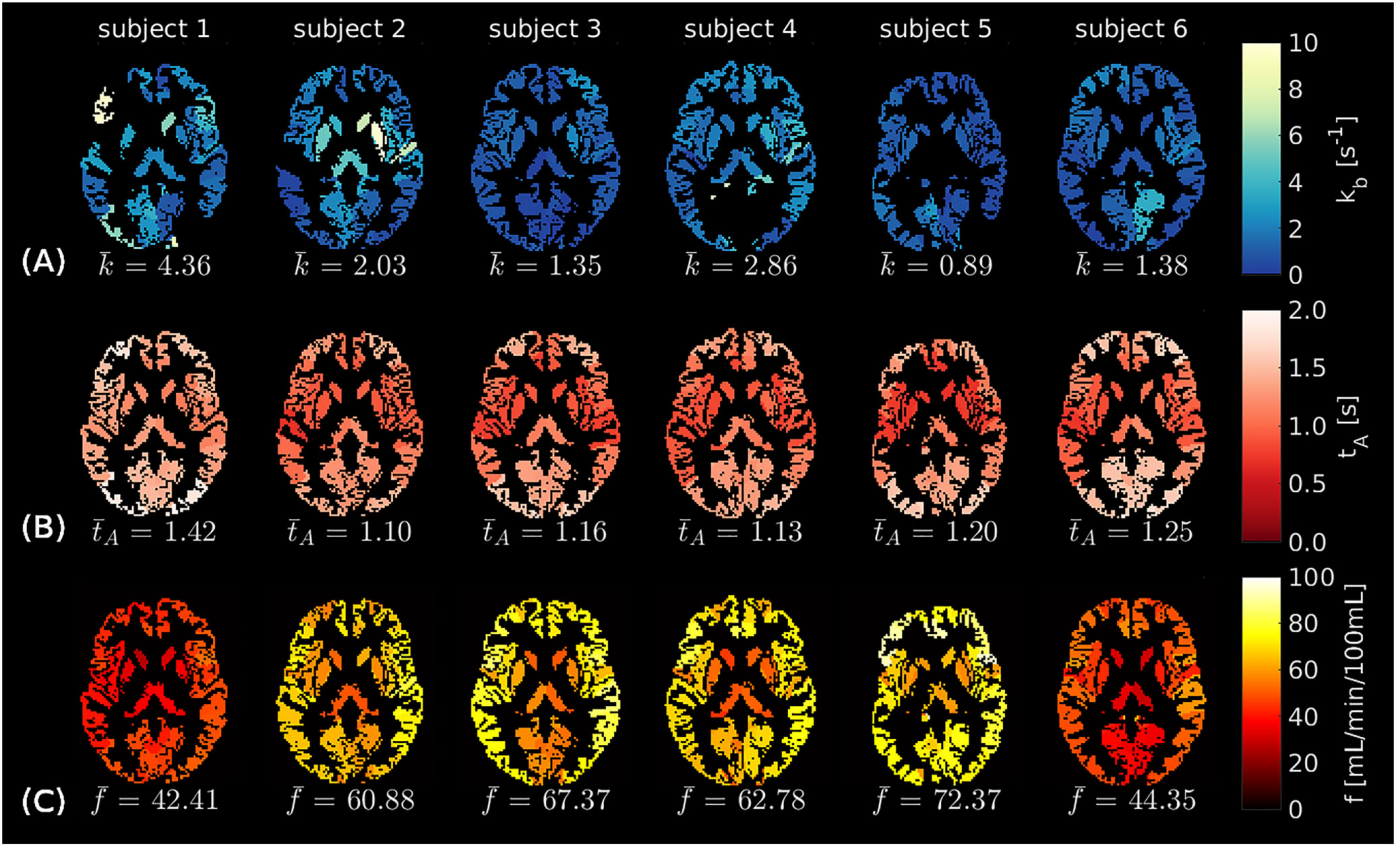
ASL regional parameter maps (PC2). (A) Exchange rate, $k_{b}$. (B) Arterial transit time, $t_{A}$. (C) Cerebral blood flow, f. All parameter values derived from the second post‐contrast data set, PC2. In all maps, black voxels represent masked white matter and CSF, as well as extreme $k_{b}$ fits (i.e., $k_{b}<0$ s^−1^ or $k_{b}>10$ s^−1^). Parameter values averaged over the ROIs ( $\bar{k}$, ${\bar{t}}_{A}$, $\bar{f}$) are displayed for each volunteer.

Figure 7 shows regional parameter maps derived from the PC2 data for all six subjects.

**FIGURE 8:**
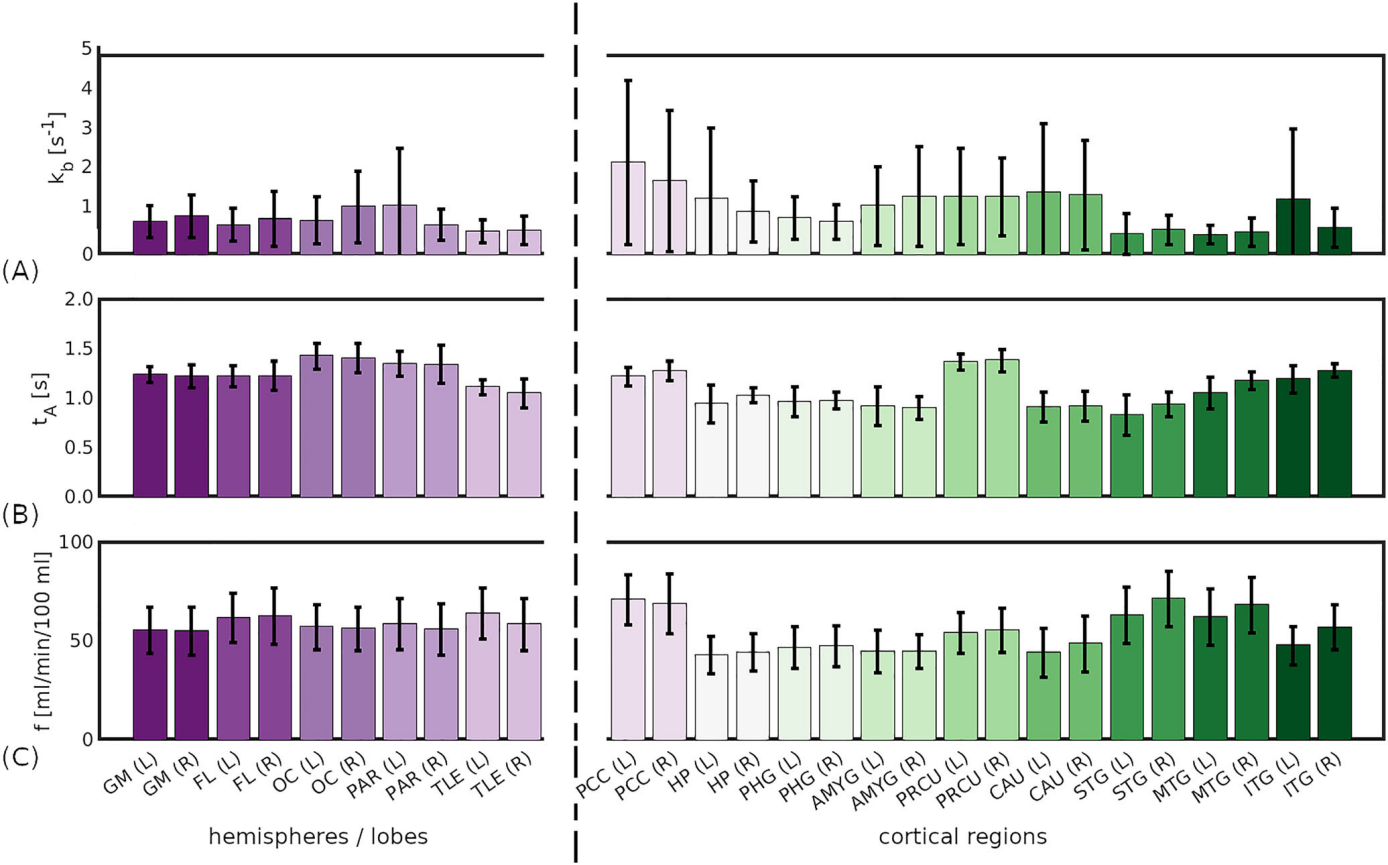
Mean parameter values across subjects in selected regions (PC2). (A) Exchange rate, $k_{b}$. (B) Arterial transit time, $t_{A}$. (C) Cerebral blood flow, f. All parameter values derived from the second post‐contrast data set, PC2. In all subplots, bar height represents the mean and error bars show the standard deviation across subjects.

Mean regional parameter estimates for a selection of 18 cortical and sub‐cortical regions (of particular relevance to dementia) are provided in Figure 8; results from all 90 regions can be found in the Supporting Information, Table S5. Good left/right hemispheric correspondence was observed in the exchange rate maps, although a few extreme fits ( $k_{b}<0$ s^−1^ or $k_{b}>10$ s^−1^) were noted in Subjects 1 and 2. Data for these subjects were acquired without the shortest PLD ( PLD=0.7 s), so fit instabilities may have arisen owing to the reduced number of data points. Averaged across subjects, the means and standard deviations of parameter values were $t_{A}=1.15\pm 0.49$ s, f=58.0±14.3 mL blood/min/100 mL tissue, $k_{b}=2.32\pm 2.49$ s^−1^.

The mean SNR across segmented regions in the subtraction image at PLD=1.5 s was 3.6 before contrast and 3.1 in the PC2 data (corresponding to SNR∼30 in the unlabelled data in simulations), indicating that the increased number of averages after contrast compensated well for the expected loss of signal. Good agreement was observed between the data and model fits before and after contrast (see Supporting Information, Figure S6).

## 5 DISCUSSION

This simulation and proof‐of‐concept study demonstrates that measurements of BBB permeability to water are feasible using CE‐ASL if accurate $T_{1}$ values can be obtained: under the influence of an intravascular GBCA, the increased difference between blood water and tissue $T_{1}$ relaxation times enables the signal contribution from intra‐ and extravascular compartments to be distinguished and $k_{b}$ to be estimated.

Identifying the optimal difference between blood water and tissue $T_{1}$ relaxation times is key to obtaining reliable water exchange estimates using this method owing to the inherent trade‐off between the reduction in $T_{1,b}^{\text{post}}$ and sensitivity to $k_{b}$ (Figure 3C). Marginal reductions in $T_{1,b}^{\text{post}}$ do not sufficiently perturb the post‐contrast signal, meaning that the relative contributions of intra‐ and extravascular compartments remain difficult to separate and sensitivity is correspondingly low. Conversely, extreme reductions in $T_{1,b}^{\text{post}}$ lead to a vanishing difference signal as fast recovery of the labelled spins relative to the ATT negates the effect of the inversion, rendering the post‐contrast signal equivalent to the control data. It was shown using simulations that moderate reductions in $T_{1,b}^{\text{post}}$ best enabled water exchange measurements, and, moreover, that a range of $T_{1,b}^{\text{post}}$ values presented similar capacities to reliably estimate $k_{b}$. Practically, this allows for flexibility in protocol design, as precise timings of the post‐contrast ASL acquisition relative to GBCA administration—as well as precise administration of the GBCA dose itself—are not critical; however, increasing the number of signal averages for lower $T_{1,b}^{\text{post}}$ values may be prudent as the difference signal will be smaller and more susceptible to noise. Simulations showed that even a quarter dose (0.025 mmol/kg) could provide the optimal $T_{1,b}^{\text{post}}$ (Figure 6), which has reduced safety concerns compared with a full dose.

The sensitivity analysis indicated that more precise $k_{b}$ estimates can be expected at lower exchange rates (Figure 3C), meaning that CE‐ASL is best suited to probing early, subtle damage. Monte Carlo simulations supported this finding and quantified the measurement precision of model parameters in terms of ground truth $k_{b}$ (Figure 5A–C). The accuracy and precision of fitted parameters was also quantified as a function of SNR (Figure 5D–I). CBF and ATT were estimated at the voxel level with acceptable precision at realistic noise levels in synthetic data.

Reliable estimates of $k_{b}$ at the voxel level proved unfeasible in simulations, with a measurement precision approaching CoV∼190% (Figure 5G): inherently low SNR data combined with low sensitivity of the model to $k_{b}$ (relative to the CBF and ATT) renders $k_{b}$ a challenging parameter to fit. Voxel‐wise fits in vivo (see Supporting Information, Figure S7) corresponded to the simulation predictions, with a number of extreme fits apparent. ROI analyses are valuable in this instance as the SNR effectively increases as the square root of the number of voxels, reducing random measurement errors and making regional $k_{b}$ estimates more reliable (Figure 7A). Practically, it should be noted that an upper limit on the ROI size used for regional analysis is likely to exist owing to variability in ATT, CBF and potentially $k_{b}$ across the brain, particularly in disease.

Regional in vivo $k_{b}$ measurements (Figure 8) were in agreement with literature values. The mean value across all segmented ROIs and subjects was $k_{b}=2.32$ s^−1^; previous studies have reported average GM values in the range 0.63–3.68 s^−1^.^13^ There was also good agreement in regional values between hemispheres, suggesting that physiologically plausible $k_{b}$ values can be obtained using CE‐ASL. CBF was notably lower in two subjects, although in line with published values^32^, ^44^, ^45^ and reports of high inter‐subject variability.^46^ Age and gender may also explain this finding: one of the two subjects was the only male and one was older, and lower CBF has been reported in both demographics.^47^, ^48^

There are two limiting factors in the Monte Carlo simulations in this study. First, regional variations in ATT observed in vivo were not modelled in the simulations: regions with $t_{A}>1.5$ s (i.e., ATT longer than the post‐contrast PLD) are likely to have lower precision as the complete bolus may not have arrived at the voxel at the time of imaging. Post hoc evaluation of the sequence sensitivity and the accuracy and precision of $k_{b}$ estimates confirmed this: while high accuracy was maintained regardless of ATT, longer ATTs incurred lower sensitivity (reduced by approximately 35% at $t_{A}=1.5$ s compared with $t_{A}=1.2$ s) and subsequently lower precision (IQR increased by approximately 65% at $t_{A}=1.5$ s compared with $t_{A}=1.2$ s) (see Supporting Information, Figure S8). Acquiring data at multiple PLDs post‐contrast may increase sensitivity for regions with longer ATT (or potentially with variations in $T_{1,b}$, as might occur owing to haematocrit changes in sickle cell disease^49^) and should be evaluated in future work; a single post‐contrast PLD=1.5 s was chosen here to allow time for multiple signal averages. Second, alterations in $T_{1,e}$ were not modelled in the simulations: contrast agent leakage into the extravascular space acts to decrease the difference between the post‐contrast blood water and tissue $T_{1}$ times, making the separation of intra‐ and extravascular signal components more challenging. The sensitivity analysis confirmed this, showing that, as $T_{1,e}^{\text{post}}\to T_{1,b}^{\text{post}},k_{b}$ was estimated with increasingly poorer precision. In our proof‐of‐concept study a reduction of about 7% in tissue $T_{1}$ was observed; however, as this is the total tissue value and includes the blood component, the reduction in $T_{1,b}$ post‐contrast probably explains the majority of this decrease. Moreover, the decrease was consistent across subjects and so does not suggest leakage due to pathology.

Nonetheless, this is a minor limitation of the technique, and raises the more general point that $T_{1,e}$ is not independent of $k_{b}$. This was also not modelled in simulations and should be considered in future studies. In the in vivo data, adding vascular crushers to the acquisition may improve estimation of the ATT and CBF: without vascular crushers, the signal at short PLDs may contain contributions from large vessels, leading to shorter ATT and higher CBF; however, as no exchange is expected to occur in large vessels, $k_{b}$ is unlikely to be affected.

The primary limitation of CE‐ASL is the accuracy required in $T_{1}$ measurements (Figure 4). Similar systematic errors in both $T_{1,b}^{\text{pre}}$ and $T_{1,b}^{\text{post}}$ may mitigate error propagation into $k_{b}$ to some extent as opposing effects are introduced (see Supporting Information, Figure S9); however, particularly for $T_{1,b}^{\text{post}}$, where sensitivity to $k_{b}$ and therefore error propagation is greatest, small errors can introduce significant biases into $k_{b}$ measurements. The potential effect on in vivo parameter estimates arising from $T_{1,b}$ biases was explored post hoc by perturbing the measured $T_{1,b}^{\text{pre}}$ and $T_{1,b}^{\text{post}}$ by ±10% and re‐fitting the model (see Supporting Information, Figure S10). Mean fitted parameter values varied according to the trends predicted in Figure S9, with $k_{b}$ increased on average by 100% for $T_{1,b}$ adjusted 10% lower and reduced by 44% for $T_{1,b}$ adjusted 10% higher than the measured value. It must also be considered that inter‐subject variability in $T_{1,b}$ can be introduced depending on haematocrit levels and oxygen extraction fraction,^50^ which further emphasizes the need for reliable individual $T_{1,b}$ mapping; however, regional $k_{b}$ variations within a subject may still be identified. Finally, clinical conditions in which key assumptions of the model are violated—for example arteriovenous malformations, where the passage of blood into the microvasculature is disrupted and the assumption of no outflow no longer holds—must be treated cautiously.

Given current clinical capabilities, these $T_{1}$ accuracy requirements limit the utility of the CE‐ASL technique at this point. Assuming a situation where $T_{1}$ values are accurate enough, a clinically practical application of CE‐ASL would be in conjunction with conventional DCE‐MRI studies: ASL data acquired before and after a DCE‐MRI protocol could utilize the residual effects of the GBCA to obtain the optimally shortened $T_{1,b}^{\text{post}}$ needed for CE‐ASL imaging, and dose calculations suggest that the time to optimal $T_{1,b}^{\text{post}}$ following a full‐dose injection could make this approach feasible (32 min to reach the lower bound $T_{1,b}^{\text{post}}=0.6$ s). In cases where BBB damage is minor and DCE‐MRI does not show significant uptake of the contrast agent in the tissue,^3^ concomitant acquisition of CE‐ASL data could provide a complementary indication of subtle BBB breakdown. However, it is necessary to reduce the dependence of $k_{b}$ estimates on measured $T_{1}$ values for this to be clinically viable.

## Supporting information



nbm5009‐sup‐0001‐Supporting Information.pdf

## Data Availability

The data that support the findings of this study are available on request from the corresponding author. The data are not publicly available due to privacy or ethical restrictions.

## SENSITIVITY FUNCTIONS

### 1

The sensitivity functions are defined as the partial derivative of the signal model with respect to $k_{b}$. The shape and magnitude of the functions therefore indicate the sensitivity of the signal model to changes in $k_{b}$; the sign of the functions indicates whether a change in $k_{b}$ induces an increase or decrease in signal intensity. The sensitivity functions for the CASL model in Equation (4) are

(A1)
$$\frac{\partial \Delta M\left(t\right)}{\partial k_{b}}=\left\{\begin{array}{ll} 0 & t<t_{A}\\ \begin{array}{cc} af\exp\left(-R_{1,b}t_{A}\right) & \left\{\frac{t^{\prime }\exp\left(-Jt^{\prime }\right)}{J}-\frac{1-\exp\left(-Jt^{\prime }\right)}{J^{2}}+\frac{J\left[1-\exp\left(-R_{1,e}t^{\prime }\right)\right]-R_{1,e}\left[1-\exp\left(-Jt^{\prime }\right)\right]}{JR_{1,e}\left(J-R_{1,e}\right)}\right.\\ & -\frac{k_{b}J\left[1-\exp\left(-R_{1,e}t^{\prime }\right)\right]-k_{b}R_{1,e}\left[1-\exp\left(-Jt^{\prime }\right)\right]}{JR_{1,e}{\left(J-R_{1,e}\right)}^{2}}\\ & -\frac{k_{b}J\left[1-\exp\left(-R_{1,e}t^{\prime }\right)\right]-k_{b}R_{1,e}\left[1-\exp\left(-Jt^{\prime }\right)\right]}{J^{2}R_{1,e}\left(J-R_{1,e}\right)}\\ & \left.-\frac{k_{b}\left[\exp\left(-R_{1,e}t^{\prime }\right)+R_{1,e}t^{\prime }\exp\left(-Jt^{\prime }\right)-1\right]}{JR_{1,e}\left(J-R_{1,e}\right)}\right\} \end{array} & t_{A}\leq t\leq t_{A}+t_{L}\\ \begin{array}{cc} -af\exp\left(-R_{1,b}t_{A}\right) & \left\{\exp\left(-Jt^{\prime }\right)\left[\exp\left(Jt_{L}\right)-1\right]\left[\frac{1}{J^{2}}+\frac{1}{J\left(J-R_{1,e}\right)}-\frac{k_{b}}{J{\left(J-R_{1,e}\right)}^{2}}-\frac{k_{b}}{J^{2}\left(J-R_{1,e}\right)}\right]\right.\\ & -t_{L}\exp\left(Jt_{L}\right)\exp\left(-Jt^{\prime }\right)\left[\frac{1}{J}-\frac{k_{b}}{J\left(J-R_{1,e}\right)}\right]\\ & +t^{\prime }\exp\left(-Jt^{\prime }\right)\left[\exp\left(Jt_{L}\right)-1\right]\left[\frac{1}{J}-\frac{k_{b}}{J\left(J-R_{1,e}\right)}\right]\\ & -\frac{\exp\left(-R_{1,e}t^{\prime }\right)\left[\exp\left(R_{1,e}t_{L}\right)-1\right]}{R_{1,e}\left(J-R_{1,e}\right)}\\ & \left.+\frac{k_{b}\exp\left(-R_{1,e}t^{\prime }\right)\left[\exp\left(R_{1,e}t_{L}\right)-1\right]}{R_{1,e}{\left(J-R_{1,e}\right)}^{2}}\right\} \end{array} & t>t_{A}+t_{L} \end{array}\right..$$

where $t_{L}$ is the labelling time, $t_{A}$ is the arterial transit time, f is the cerebral blood flow, α is the inversion efficiency of the labelling, $R_{1,e}={1/T}_{1,e}$, $R_{1,b}={1/T}_{1,b}$, $J=k_{b}+R_{1,b}$ and $t\prime =t-t_{A}$.
