# Supplementary material for: Blood–brain barrier water exchange measurements using contrast‐enhanced ASL
Source: NMR Biomed. 2023 Sep 4;36(11):e5009. doi: 10.1002/nbm.5009 (PMC10909569; doi:10.1002/nbm.5009)
Supplement: Supplementary file 1 — nbm5009‐sup‐0001‐Supporting Information.pdf [file NBM-36-e5009-s001.pdf]

Supporting information S1: ROIs used for blood $T_{1,b}$ estimation

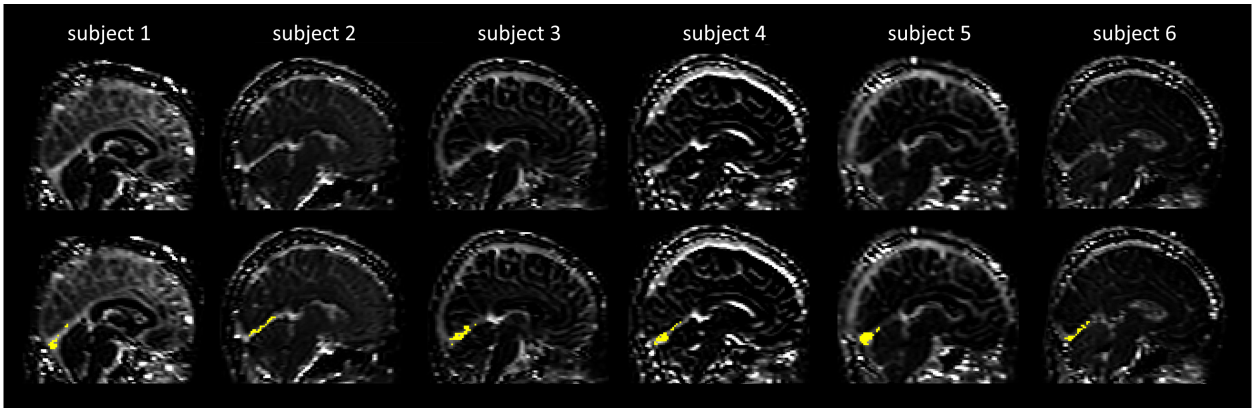

Figure S1. ROIs for blood $T_{1,b}$ estimation.

The top row shows the subtraction images (post-contrast T_1 map subtracted from the pre-contrast map) for each subject; the bottom row highlights (in yellow) the ROIs defined in the sagittal sinus and straight sinus.

Supporting information S2: extreme fits (simulated data)

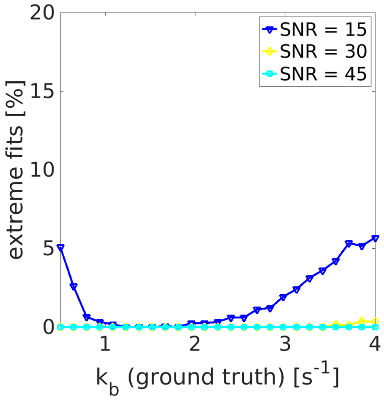

Figure S2. Number of extreme fits (simulated data).

The number of extreme fits (defined as any parameter having hit a bound) in the Monte Carlo accuracy and precision simulations as a function of the exchange rate, k_b. Ground truth parameter values were: $k_b = 2.65\,\mathrm{s}^{-1}$, $f = 60\,\mathrm{ml\,blood\,/\,min\,/\,100\,ml\,tissue}$, $t_A = 1.2\,\mathrm{s}$, $T_{1,b}^{pre} = 1.65\,\mathrm{s}$, $T_{1,b}^{post} = 0.8\,\mathrm{s}$, $T_{1,e} = 1.5\,\mathrm{s}$, 5 pre-contrast PLDs between $0.9 - 2.1\,\mathrm{s}$, 1 post-contrast PLD $= 1.5\,\mathrm{s}$, $t_L = 2\,\mathrm{s}$, $\lambda = 0.9$ and $\alpha = 0.85$.

Supporting information S3: representative ASL subtraction images and tissue T_1 maps pre- and post-contrast

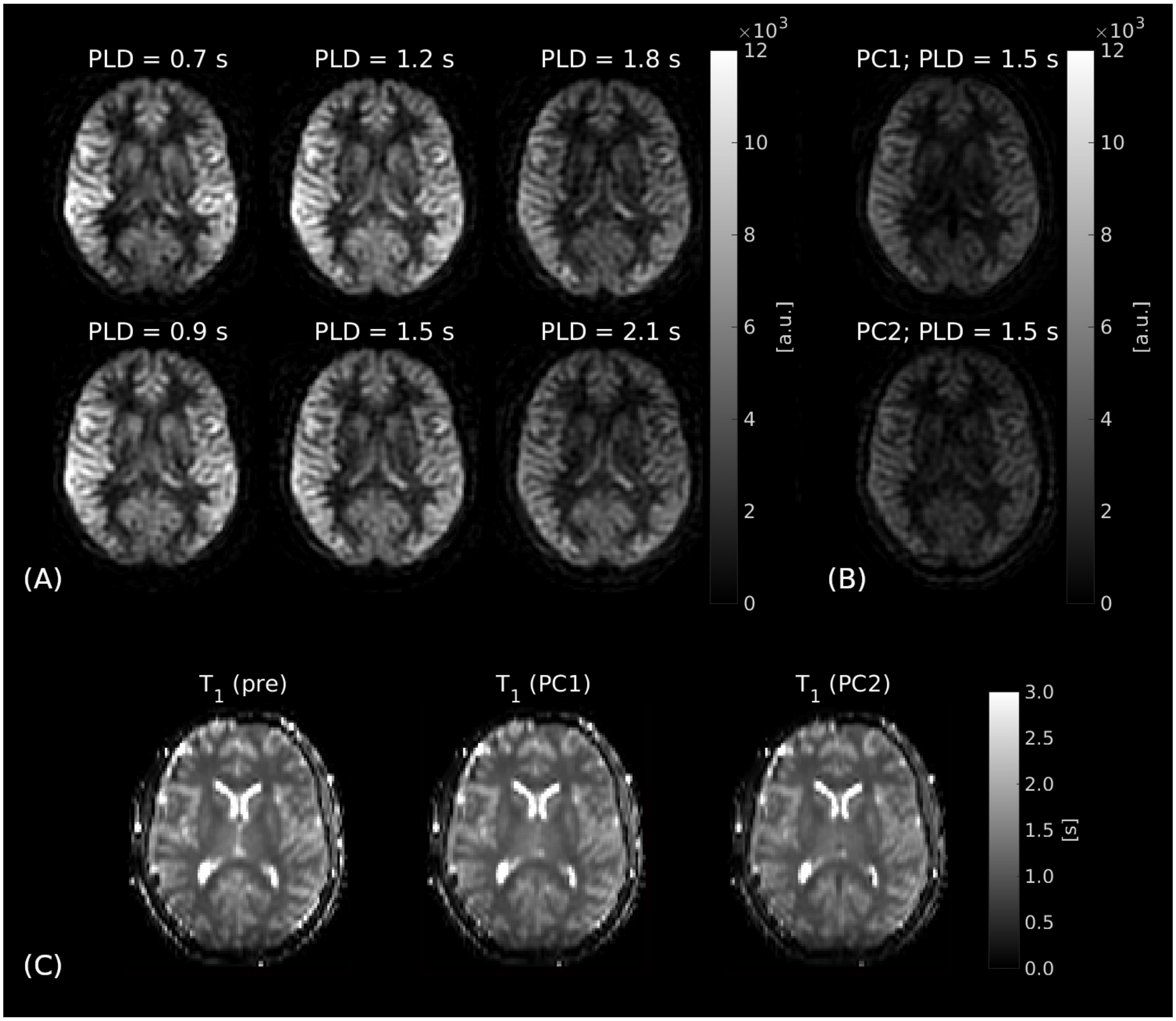

Figure S3. Representative ASL data and tissue T_1 maps.

(A). ASL subtraction images pre-contrast at all post-labelling delay (PLD) times. The SNR at PLD = 1.5 s was 3.9. **(B)**. ASL subtraction data at the single PLD for the first (top; PC1) and second (bottom; PC2) post-contrast acquisitions. The SNR was 3.6 for PC1 and 3.1 for PC2. Good grey:white matter contrast remained visible following both contrast agent injections. **(C)**. Tissue T_1 maps pre- and post-contrast. Minimal difference was observed, indicating that the contrast agent remained in the intravascular space.

Supporting information S4: results from PC1

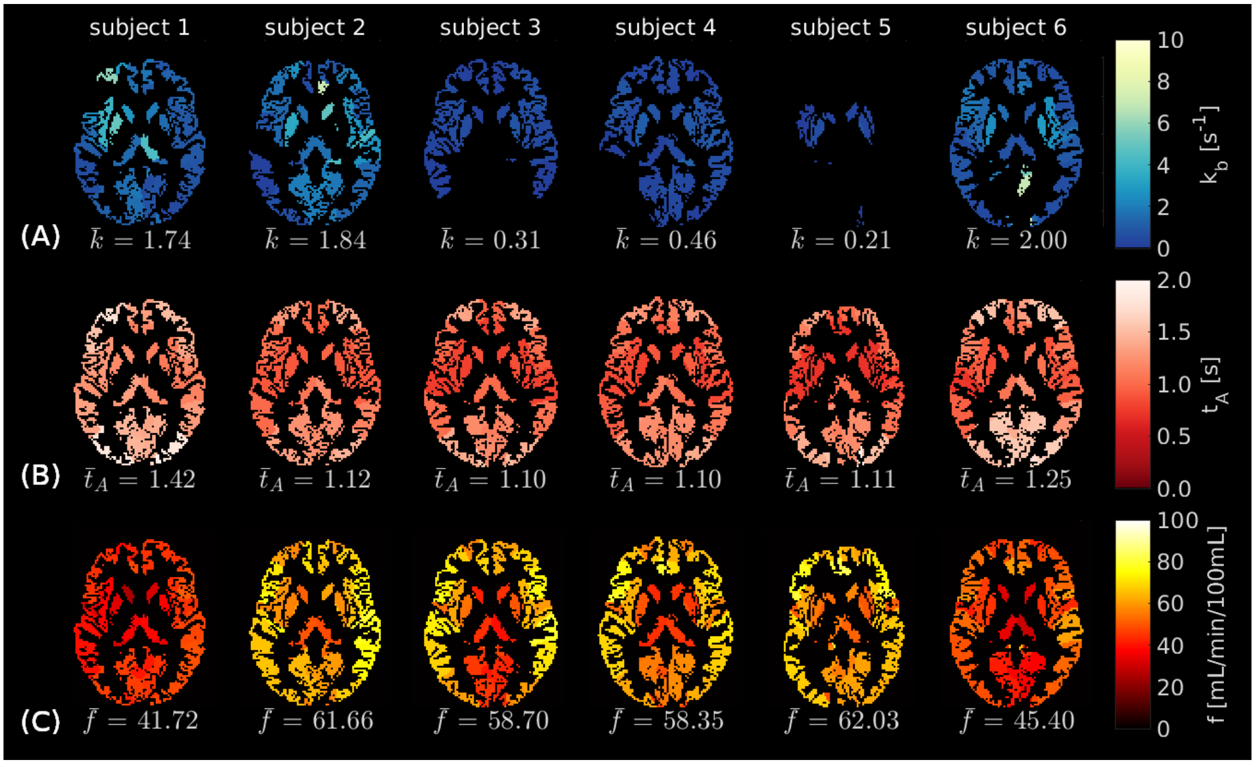

Figure S4.1. ASL regional parameter maps (PC1).

(A). Exchange rate, k_b. **(B)**. Arterial transit time, t_A. **(C)**. Cerebral blood flow, f. All parameter values derived from the first post-contrast data set, PC1. In all maps, black voxels represent masked white matter and CSF, as well as extreme k_b fits (i.e. $k_b < 0\,\mathrm{s}^{-1}$ or $k_b > 10\,\mathrm{s}^{-1}$). Parameter values averaged over the ROIs ($\bar{k}$, $\bar{t_A}$, $\bar{f}$) are displayed for each volunteer. It is possible that measurement errors owing to rapid $T_{1,b}^{post}$ recovery at the beginning of the T_1 mapping sequence (which was acquired approximately 3 minutes after each contrast agent injection; see also Figure 6) contributed to increased fit instabilities (indicated by the greater proportion of extreme fits) relative to the second post-contrast (PC2) data.

Table S4.2. ASL regional parameter fits (PC1).

Values are the mean and standard deviation across subjects of regional fits using the first post-contrast data, PC1. GM = grey matter; FL = frontal lobe; OC = occipital lobe; PAR = parietal lobe; TLE = temporal lobe; PrcG = precentral gyrus; SFG = superior frontal gyrus; OFGsup = superior orbitofrontal gyrus; MFG = middle frontal gyrus; OFGmid = middle orbitofrontal gyrus; IFGop = inferior frontal gyrus (opercular part); IFGtr = inferior frontal gyrus (triangular part); OFGinf = inferior orbitofrontal gyrus; ROL = rolandic operculum; SMA = supplementary motor area; OLF = olfactory cortex; SFGmed = superior frontal gyrus (medial part); OFGmid = orbitofrontal gyrus (middle part); REC = rectus; INS = insula; ACC = anterior cingulate cortex; MCC = middle cingulate cortex; PCC = posterior cingulate cortex; HP = hippocampus; PHG = parahippocampal gyrus; AMYG = amygdala; CAL = calcarine; CUN = cuneus; LING = lingual gyrus; SOG = superior occipital gyrus; MOG = middle occipital gyrus; IOG = inferior occipital gyrus; FFG = fusiform gyrus; PocG = postcentral gyrus; SPG = superior parietal gyrus; IPL = inferior parietal lobule; SMG = supramarginal gyrus; ANG = angular gyrus; PRCU = precuneus; PCL = paracentral lobule; CAU = caudate; PUT = putamen; PAL = pallidum; THAL = thalamus; HES = Heschl gyrus; STG = superior temporal gyrus; TPsup = temporal pole (superior part); MTG = middle temporal gyrus; TPmid = temporal pole (middle part); ITG = inferior temporal gyrus; L = left; R = right.

Region name	No. voxels	f [ml / min / 100 ml]	t_A [s]	k_b [s^{-1}]
GM (L)	55805	51.29 ± 9.81	1.21 ± 0.12	0.90 ± 0.95
GM (R)	46429	51.53 ± 8.80	1.20 ± 0.13	0.82 ± 0.84
FL (L)	12154	58.22 ± 10.31	1.20 ± 0.12	0.69 ± 0.70
FL (R)	11744	58.98 ± 10.42	1.20 ± 0.15	0.92 ± 0.86
OC (L)	6678	51.55 ± 7.15	1.38 ± 0.15	0.84 ± 1.15
OC (R)	7591	51.28 ± 8.28	1.37 ± 0.17	0.69 ± 1.22
PAR (L)	4287	54.03 ± 9.38	1.31 ± 0.14	0.69 ± 0.89
PAR (R)	4561	52.19 ± 9.05	1.32 ± 0.18	0.48 ± 0.59
TLE (L)	7046	59.76 ± 10.80	1.09 ± 0.14	0.71 ± 0.69
TLE (R)	6643	56.33 ± 10.48	1.04 ± 0.13	0.36 ± 0.43
PrcG (L)	1379	54.74 ± 8.67	1.34 ± 0.24	0.95 ± 1.06
PrcG (R)	1212	53.75 ± 9.02	1.32 ± 0.13	0.65 ± 0.85
SFG (L)	1366	52.42 ± 8.86	1.38 ± 0.13	1.07 ± 1.21
SFG (R)	1696	54.20 ± 8.10	1.36 ± 0.13	0.39 ± 0.43
OFGsup (L)	494	53.05 ± 9.53	1.17 ± 0.16	0.91 ± 0.73
OFGsup (R)	511	50.10 ± 7.31	1.18 ± 0.11	0.59 ± 0.67
MFG (L)	2142	60.59 ± 11.72	1.43 ± 0.19	1.43 ± 2.09
MFG (R)	2293	61.15 ± 10.75	1.40 ± 0.11	0.72 ± 0.75
OFGmid (L)	428	59.65 ± 10.55	1.28 ± 0.16	1.20 ± 1.02

Region name	No. voxels	f	t_A	k_b
OFGmid (R)	471	60.45 ± 10.49	1.26 ± 0.15	1.21 ± 1.19
IFGop (L)	439	69.25 ± 15.39	1.07 ± 0.17	1.04 ± 1.11
IFGop (R)	615	69.58 ± 11.37	1.10 ± 0.12	0.60 ± 0.63
IFGtr (L)	1024	65.85 ± 12.53	1.15 ± 0.18	2.44 ± 3.49
IFGtr (R)	808	65.35 ± 11.45	1.09 ± 0.14	0.68 ± 0.66
OFGinf (L)	856	64.03 ± 12.86	1.05 ± 0.17	2.41 ± 3.51
OFGinf (R)	768	61.14 ± 9.75	0.94 ± 0.20	1.01 ± 0.76
ROL (L)	514	47.87 ± 7.93	0.93 ± 0.19	0.75 ± 0.69
ROL (R)	689	54.71 ± 11.23	0.97 ± 0.11	0.88 ± 0.64
SMA (L)	905	54.88 ± 10.39	1.16 ± 0.16	0.54 ± 0.47
SMA (R)	1016	52.06 ± 8.11	1.18 ± 0.19	0.90 ± 0.94
OLF (L)	187	55.23 ± 11.35	0.84 ± 0.17	1.48 ± 1.54
OLF (R)	189	52.79 ± 10.15	0.89 ± 0.14	1.50 ± 1.54
SFGmed (L)	1162	60.22 ± 11.89	1.07 ± 0.14	0.53 ± 1.07
SFGmed (R)	983	58.58 ± 8.63	1.12 ± 0.14	0.71 ± 0.67
OFGmed (L)	370	70.95 ± 14.19	1.00 ± 0.18	0.67 ± 0.56
OFGmed (R)	463	66.54 ± 9.63	1.00 ± 0.15	1.13 ± 1.63
REC (L)	478	67.90 ± 12.05	0.93 ± 0.16	0.48 ± 0.52
REC (R)	440	59.51 ± 8.32	0.96 ± 0.13	0.78 ± 1.06
INS (L)	1243	54.92 ± 9.73	0.91 ± 0.18	1.28 ± 1.28
INS (R)	1097	59.46 ± 10.43	0.85 ± 0.13	1.24 ± 1.08
ACC (L)	812	68.02 ± 13.40	0.90 ± 0.13	0.67 ± 0.90
ACC (R)	753	63.87 ± 11.87	0.94 ± 0.15	1.74 ± 2.52
MCC (L)	1095	58.77 ± 9.66	1.08 ± 0.10	1.08 ± 1.23
MCC (R)	1292	60.13 ± 11.17	1.07 ± 0.17	1.84 ± 2.16
PCC (L)	192	66.03 ± 11.41	1.19 ± 0.08	2.21 ± 3.77
PCC (R)	110	63.30 ± 10.97	1.24 ± 0.13	2.20 ± 4.68
HP (L)	614	40.63 ± 11.61	0.92 ± 0.25	0.80 ± 3.58
HP (R)	530	41.72 ± 7.83	0.99 ± 0.13	1.38 ± 1.48
PHG (L)	555	43.62 ± 9.02	0.93 ± 0.16	1.04 ± 1.32
PHG (R)	698	44.33 ± 8.63	0.94 ± 0.11	0.99 ± 1.50
AMYG (L)	174	42.24 ± 9.16	0.90 ± 0.18	1.71 ± 2.19
AMYG (R)	187	42.74 ± 6.75	0.89 ± 0.15	2.67 ± 3.05
CAL (L)	1266	52.19 ± 10.28	1.31 ± 0.20	2.22 ± 3.78
CAL (R)	1010	50.63 ± 10.03	1.30 ± 0.17	2.06 ± 4.71
CUN (L)	715	47.04 ± 6.92	1.37 ± 0.14	1.18 ± 3.66
CUN (R)	723	49.65 ± 7.01	1.43 ± 0.20	1.72 ± 2.96
LING (L)	1253	47.05 ± 8.79	1.22 ± 0.22	2.31 ± 4.12

Region name	No. voxels	f	t_A	k_b
LING (R)	1296	49.17 ± 9.87	1.26 ± 0.14	1.62 ± 4.06
SOG (L)	549	45.56 ± 6.26	1.56 ± 0.16	0.28 ± 2.58
SOG (R)	576	53.22 ± 8.08	1.66 ± 0.24	0.83 ± 0.88
MOG (L)	1716	58.47 ± 8.97	1.45 ± 0.21	0.24 ± 0.70
MOG (R)	1035	58.76 ± 7.95	1.52 ± 0.14	0.28 ± 0.57
IOG (L)	542	57.00 ± 11.60	1.44 ± 0.21	0.32 ± 0.62
IOG (R)	495	55.36 ± 10.54	1.46 ± 0.17	0.17 ± 0.47
FFG (L)	1562	39.61 ± 7.98	1.18 ± 0.21	1.23 ± 1.34
FFG (R)	1635	38.92 ± 8.66	1.20 ± 0.07	1.35 ± 1.78
PoCG (L)	1473	52.10 ± 12.63	1.30 ± 0.19	0.53 ± 1.55
PoCG (R)	1341	48.30 ± 8.02	1.31 ± 0.11	0.51 ± 0.69
SPG (L)	704	43.17 ± 5.88	1.58 ± 0.21	0.82 ± 1.47
SPG (R)	620	42.77 ± 7.24	1.60 ± 0.19	0.79 ± 0.82
IPL (L)	1165	51.80 ± 11.24	1.35 ± 0.25	0.68 ± 0.97
IPL (R)	583	54.35 ± 9.02	1.37 ± 0.14	0.75 ± 0.88
SMG (L)	599	57.87 ± 11.70	1.11 ± 0.20	0.39 ± 0.50
SMG (R)	921	62.10 ± 11.52	1.10 ± 0.11	0.82 ± 0.87
ANG (L)	621	58.81 ± 10.25	1.29 ± 0.15	0.46 ± 0.69
ANG (R)	822	59.76 ± 8.74	1.31 ± 0.11	0.73 ± 1.07
PRCU (L)	1551	48.65 ± 5.14	1.32 ± 0.10	0.76 ± 1.11
PRCU (R)	1544	50.53 ± 6.80	1.34 ± 0.14	1.46 ± 2.01
PCL (L)	420	41.54 ± 5.21	1.29 ± 0.11	0.46 ± 1.92
PCL (R)	287	43.22 ± 6.65	1.34 ± 0.15	0.51 ± 0.71
CAU (L)	545	41.15 ± 10.01	0.91 ± 0.24	1.19 ± 0.92
CAU (R)	584	45.39 ± 12.31	0.91 ± 0.15	1.50 ± 1.59
PUT (L)	674	48.80 ± 10.08	0.87 ± 0.17	2.18 ± 1.70
PUT (R)	690	47.33 ± 6.74	0.92 ± 0.13	2.74 ± 3.35
PAL (L)	71	49.80 ± 8.53	0.89 ± 0.20	2.21 ± 2.58
PAL (R)	67	50.76 ± 9.73	0.85 ± 0.13	1.52 ± 2.62
THAL (L)	357	44.20 ± 7.20	1.10 ± 0.11	0.54 ± 0.79
THAL (R)	399	42.25 ± 7.78	1.12 ± 0.11	1.03 ± 1.67
HES (L)	136	62.60 ± 17.52	0.84 ± 0.24	0.55 ± 0.74
HES (R)	139	62.10 ± 10.66	0.86 ± 0.12	1.40 ± 1.41
STG (L)	1142	61.08 ± 11.45	0.87 ± 0.18	0.33 ± 0.65
STG (R)	1474	67.33 ± 11.97	0.91 ± 0.14	0.93 ± 0.84
TPsup (L)	451	57.33 ± 11.68	0.85 ± 0.13	0.55 ± 0.44
TPsup (R)	515	57.92 ± 10.96	0.87 ± 0.18	1.16 ± 1.22
MTG (L)	2696	60.07 ± 12.00	1.06 ± 0.15	0.24 ± 0.79

Region name	No. voxels	f	t_A	k_b
MTG (R)	2427	63.24 ± 10.77	1.15 ± 0.13	0.52 ± 0.54
TPmid (L)	342	50.72 ± 9.68	1.03 ± 0.13	1.40 ± 1.55
TPmid (R)	482	49.61 ± 6.32	1.00 ± 0.16	1.23 ± 1.35
ITG (L)	1876	45.22 ± 6.99	1.18 ± 0.13	0.51 ± 0.72
ITG (R)	2009	52.48 ± 9.77	1.23 ± 0.10	0.64 ± 0.67

Supporting information S5: regional ASL parameter fits (PC2)

Table S5. ASL regional parameter fits (PC2).
Values are the mean and standard deviation across subjects of regional fits using the second post-contrast data, PC2. GM = grey matter; FL = frontal lobe; OC = occipital lobe; PAR = parietal lobe; TLE = temporal lobe; PrcG = precentral gyrus; SFG = superior frontal gyrus; OFGsup = superior orbitofrontal gyrus; MFG = middle frontal gyrus; OFGmid = middle orbitofrontal gyrus; IFGop = inferior frontal gyrus (opercular part); IFGtr = inferior frontal gyrus (triangular part); OFGinf = inferior orbitofrontal gyrus; ROL = rolandic operculum; SMA = supplementary motor area; OLF = olfactory cortex; SFGmed = superior frontal gyrus (medial part); OFGmid = orbitofrontal gyrus (middle part); REC = rectus; INS = insula; ACC = anterior cingulate cortex; MCC = middle cingulate cortex; PCC = posterior cingulate cortex; HP = hippocampus; PHG = parahippocampal gyrus; AMYG = amygdala; CAL = calcarine; CUN = cuneus; LING = lingual gyrus; SOG = superior occipital gyrus; MOG = middle occipital gyrus; IOG = inferior occipital gyrus; FFG = fusiform gyrus; PocG = postcentral gyrus; SPG = superior parietal gyrus; IPL = inferior parietal lobule; SMG = supramarginal gyrus; ANG = angular gyrus; PRCU = precuneus; PCL = paracentral lobule; CAU = caudate; PUT = putamen; PAL = pallidum; THAL = thalamus; HES = Heschl gyrus; STG = superior temporal gyrus; TPsup = temporal pole (superior part); MTG = middle temporal gyrus; TPmid = temporal pole (middle part); ITG = inferior temporal gyrus; L = left; R = right.

Region name	No. voxels	f [ml / min / 100 ml]	t_A [s]	k_b [s^{-1}]
GM (L)	55805	55.53 ± 12.30	1.25 ± 0.09	1.67 ± 0.84
GM (R)	46429	55.25 ± 12.75	1.23 ± 0.12	1.93 ± 1.06
FL (L)	12154	61.88 ± 12.73	1.23 ± 0.12	1.51 ± 0.91
FL (R)	11744	62.78 ± 15.11	1.23 ± 0.15	1.81 ± 1.47
OC (L)	6678	57.16 ± 12.67	1.43 ± 0.15	1.74 ± 1.12
OC (R)	7591	56.36 ± 12.16	1.41 ± 0.16	2.42 ± 1.72
PAR (L)	4287	58.56 ± 12.59	1.35 ± 0.14	2.49 ± 3.19
PAR (R)	4561	55.96 ± 12.75	1.35 ± 0.21	1.52 ± 0.82
TLE (L)	7046	64.23 ± 12.79	1.12 ± 0.08	1.18 ± 0.61
TLE (R)	6643	58.61 ± 12.69	1.05 ± 0.17	1.24 ± 0.77
PrcG (L)	1379	58.74 ± 16.53	1.37 ± 0.33	2.86 ± 3.63
PrcG (R)	1212	57.32 ± 12.20	1.35 ± 0.24	1.40 ± 1.09
SFG (L)	1366	56.18 ± 14.20	1.39 ± 0.30	1.56 ± 0.74
SFG (R)	1696	57.86 ± 13.22	1.40 ± 0.16	1.34 ± 0.84
OFGsup (L)	494	56.60 ± 16.07	1.20 ± 0.09	1.95 ± 1.33
OFGsup (R)	511	53.32 ± 7.75	1.22 ± 0.12	2.73 ± 2.87

Region name	No. voxels	f	t_A	k_b
MFG (L)	2142	64.83 ± 14.75	1.45 ± 0.32	2.82 ± 3.61
MFG (R)	2293	65.59 ± 14.69	1.42 ± 0.12	1.60 ± 0.81
OFGmid (L)	428	64.46 ± 14.86	1.32 ± 0.11	2.52 ± 1.77
OFGmid (R)	471	62.80 ± 14.10	1.28 ± 0.14	4.79 ± 3.48
IFGop (L)	439	72.95 ± 16.65	1.08 ± 0.15	2.88 ± 3.54
IFGop (R)	615	73.92 ± 14.68	1212 ± 0.12	1.86 ± 1.41
IFGtr (L)	1024	69.88 ± 17.41	1.18 ± 0.17	2.91 ± 3.20
IFGtr (R)	808	68.27 ± 13.45	1100 ± 0.14	1.84 ± 1.53
OFGinf (L)	856	68.32 ± 17.04	1.08 ± 0.20	2.70 ± 3.63
OFGinf (R)	768	62.89 ± 11.04	0.93 ± 0.27	1.82 ± 1.20
ROL (L)	514	50.75 ± 13.04	0.99 ± 0.22	2.96 ± 3.46
ROL (R)	689	58.11 ± 12.05	1.00 ± 0.15	1.80 ± 1.22
SMA (L)	905	58.35 ± 15.36	1.18 ± 0.15	1.67 ± 1.42
SMA (R)	1016	54.88 ± 13.61	1.19 ± 0.18	1.14 ± 0.69
OLF (L)	187	58.25 ± 14.52	0.87 ± 0.11	1.79 ± 1.03
OLF (R)	189	55.75 ± 14.21	0.91 ± 0.16	2.06 ± 1.31
SFGmed (L)	1162	63.80 ± 15.38	1.08 ± 0.14	1.24 ± 1.02
SFGmed (R)	983	63.10 ± 13.73	1.15 ± 0.13	1.22 ± 0.90
OFGmed (L)	370	74.18 ± 17.43	1.00 ± 0.17	1.10 ± 0.49
OFGmed (R)	463	71.02 ± 15.17	1.02 ± 0.16	1.86 ± 1.58
REC (L)	478	70.97 ± 17.53	0.93 ± 0.15	0.81 ± 0.53
REC (R)	440	62.54 ± 11.04	0.99 ± 0.12	1.89 ± 1.86
INS (L)	1243	57.39 ± 12.48	0.92 ± 0.21	2.91 ± 3.83
INS (R)	1097	61.62 ± 12.25	0.85 ± 0.15	1.80 ± 1.11
ACC (L)	812	71.08 ± 16.71	0.90 ± 0.14	0.99 ± 0.24
ACC (R)	753	67.65 ± 18.12	0.96 ± 0.17	2.82 ± 3.83
MCC (L)	1095	62.56 ± 14.11	1.10 ± 0.10	2.09 ± 0.99
MCC (R)	1292	63.89 ± 15.44	1.09 ± 0.18	2.44 ± 1.57
PCC (L)	192	71.00 ± 14.32	1.23 ± 0.09	4.66 ± 3.91
PCC (R)	110	69.02 ± 16.79	1.28 ± 0.11	3.72 ± 3.60
HP (L)	614	43.05 ± 9.19	0.95 ± 0.21	2.85 ± 3.82
HP (R)	530	44.37 ± 10.10	1.03 ± 0.08	2.18 ± 1.72
PHG (L)	555	46.72 ± 11.57	0.97 ± 0.17	1.85 ± 1.04
PHG (R)	698	47.49 ± 11.41	0.98 ± 0.09	1.66 ± 0.96
AMYG (L)	174	44.85 ± 11.66	0.92 ± 0.22	2.46 ± 2.14
AMYG (R)	187	44.83 ± 9.29	0.91 ± 0.13	2.93 ± 2.68
CAL (L)	1266	57.84 ± 13.07	1.36 ± 0.14	4.50 ± 4.28

Region name	No. voxels	f	t_A	k_b
CAL (R)	1010	56.81 ± 16.15	1.35 ± 0.13	3.82 ± 4.97
CUN (L)	715	52.83 ± 10.21	1.42 ± 0.13	3.46 ± 3.62
CUN (R)	723	55.12 ± 11.46	1.48 ± 0.19	3.11 ± 3.10
LING (L)	1253	52.14 ± 12.57	1.28 ± 0.12	4.92 ± 3.81
LING (R)	1296	55.16 ± 14.85	1.31 ± 0.12	3.42 ± 4.28
SOG (L)	549	49.96 ± 8.34	1.59 ± 0.20	1.50 ± 1.27
SOG (R)	576	58.53 ± 12.30	1.69 ± 0.28	3.98 ± 3.31
MOG (L)	1716	62.60 ± 11.65	1.49 ± 0.21	1.81 ± 2.15
MOG (R)	1035	64.05 ± 10.46	1.54 ± 0.16	2.01 ± 4.29
IOG (L)	542	60.60 ± 14.86	1.47 ± 0.25	2.75 ± 3.98
IOG (R)	495	61.03 ± 11.53	1.50 ± 0.18	1.92 ± 4.34
FFG (L)	1562	41.84 ± 10.54	1.21 ± 0.15	3.54 ± 3.42
FFG (R)	1635	42.62 ± 11.75	1.24 ± 0.06	2.67 ± 2.52
PoCG (L)	1473	55.99 ± 13.30	1.35 ± 0.24	1.56 ± 0.57
PoCG (R)	1341	52.46 ± 11.63	1.34 ± 0.14	1.40 ± 0.45
SPG (L)	704	46.51 ± 10.23	1.60 ± 0.21	3.94 ± 2.31
SPG (R)	620	45.27 ± 10.15	1.61 ± 0.22	4.56 ± 3.79
IPL (L)	1165	55.17 ± 12.85	1.38 ± 0.23	2.68 ± 3.90
IPL (R)	583	59.78 ± 12.99	1.41 ± 0.17	2.82 ± 3.96
SMG (L)	599	61.77 ± 15.25	1.14 ± 0.20	1.08 ± 0.32
SMG (R)	921	67.24 ± 14.54	1.14 ± 0.14	2.83 ± 3.91
ANG (L)	621	62.50 ± 14.26	1.31 ± 0.13	1.27 ± 0.74
ANG (R)	822	66.29 ± 13.12	1.36 ± 0.12	2.06 ± 1.93
PRCU (L)	1551	54.17 ± 11.16	1.37 ± 0.09	2.94 ± 2.36
PRCU (R)	1544	55.52 ± 12.24	1.39 ± 0.12	2.94 ± 1.74
PCL (L)	420	44.50 ± 7.98	1.32 ± 0.09	1.38 ± 1.76
PCL (R)	287	46.85 ± 10.76	1.36 ± 0.17	1.07 ± 0.94
CAU (L)	545	44.22 ± 13.49	0.92 ± 0.17	3.13 ± 3.77
CAU (R)	584	48.77 ± 15.66	0.92 ± 0.16	3.01 ± 2.86
PUT (L)	674	51.64 ± 10.90	0.88 ± 0.18	2.49 ± 1.45
PUT (R)	690	50.68 ± 9.07	0.95 ± 0.13	3.53 ± 3.44
PAL (L)	71	52.74 ± 11.07	0.90 ± 0.19	3.62 ± 2.56
PAL (R)	67	54.81 ± 12.35	0.87 ± 0.15	1.98 ± 1.30
THAL (L)	357	48.35 ± 11.15	1.13 ± 0.11	1.54 ± 1.88
THAL (R)	399	46.75 ± 11.94	1.17 ± 0.07	1.64 ± 1.72
HES (L)	136	63.70 ± 15.17	-0.74 ± 4.18	2.19 ± 4.26
HES (R)	139	65.36 ± 12.58	0.87 ± 0.11	3.11 ± 2.55
STG (L)	1142	63.24 ± 13.60	0.83 ± 0.23	1.07 ± 1.14

Region name	No. voxels	f	t_A	k_b
STG (R)	1474	71.43 ± 13.68	0.94 ± 0.11	1.26 ± 0.76
TPsup (L)	451	60.06 ± 14.99	0.85 ± 0.15	0.91 ± 0.22
TPsup (R)	515	60.52 ± 14.11	0.88 ± 0.20	1.33 ± 1.13
MTG (L)	2696	62.31 ± 14.28	1.06 ± 0.18	1.03 ± 0.52
MTG (R)	2427	68.43 ± 14.11	1.18 ± 0.08	1.13 ± 0.80
TPmid (L)	342	54.28 ± 12.58	1.06 ± 0.09	1.46 ± 0.28
TPmid (R)	482	51.26 ± 8.23	1.02 ± 0.12	1.44 ± 0.75
ITG (L)	1876	47.82 ± 9.20	1.20 ± 0.15	2.81 ± 3.87
ITG (R)	2009	57.09 ± 11.50	1.28 ± 0.07	1.37 ± 1.02

Supporting information S6: model fit quality

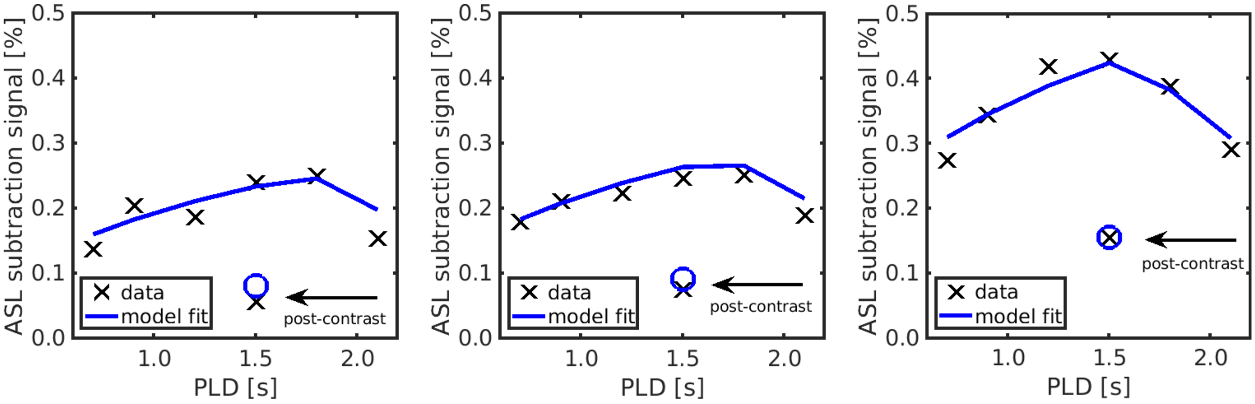

Figure S6. Model fit quality.

ASL difference signals (black) are shown for three randomly chosen voxels in a single subject overlaid with the model fits (Equation 4) (blue). Good agreement is observed between the data and model fits.

Supporting information S7: voxel-wise model fits

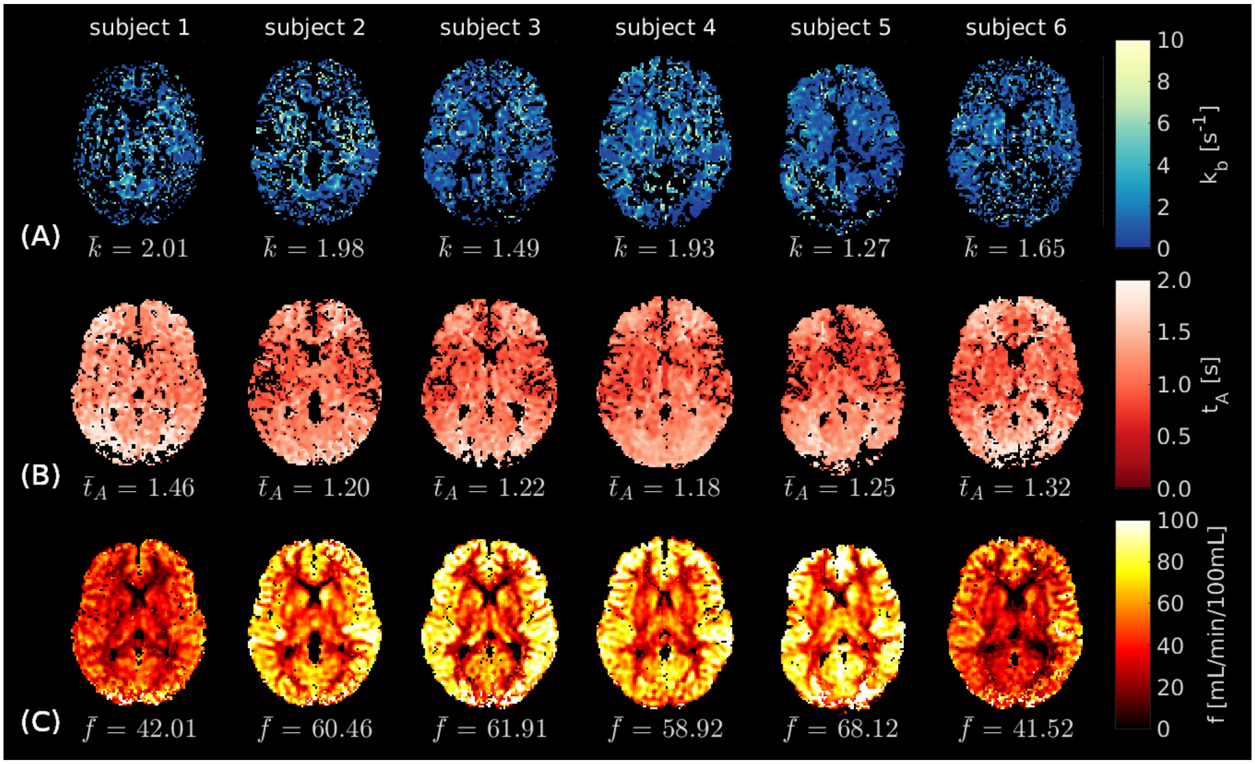

Figure S7. ASL voxel-wise parameter maps (PC2).

(A). Exchange rate, k_b. **(B)**. Arterial transit time, t_A. **(C)**. Cerebral blood flow, f. All parameter values derived from the second post-contrast data set, PC2. In all maps, black voxels represent masked CSF and extreme parameter fits ($k_b < 0\,\mathrm{s}^{-1}$ or $k_b > 10\,\mathrm{s}^{-1}$; $t_A \leq 0$; $f \leq 0$). Parameter values averaged over all voxels ($\bar{k}$, $\bar{t_A}$, $\bar{f}$) are displayed for each volunteer.

Supporting information S8: effect of arterial transit time (ATT) variation

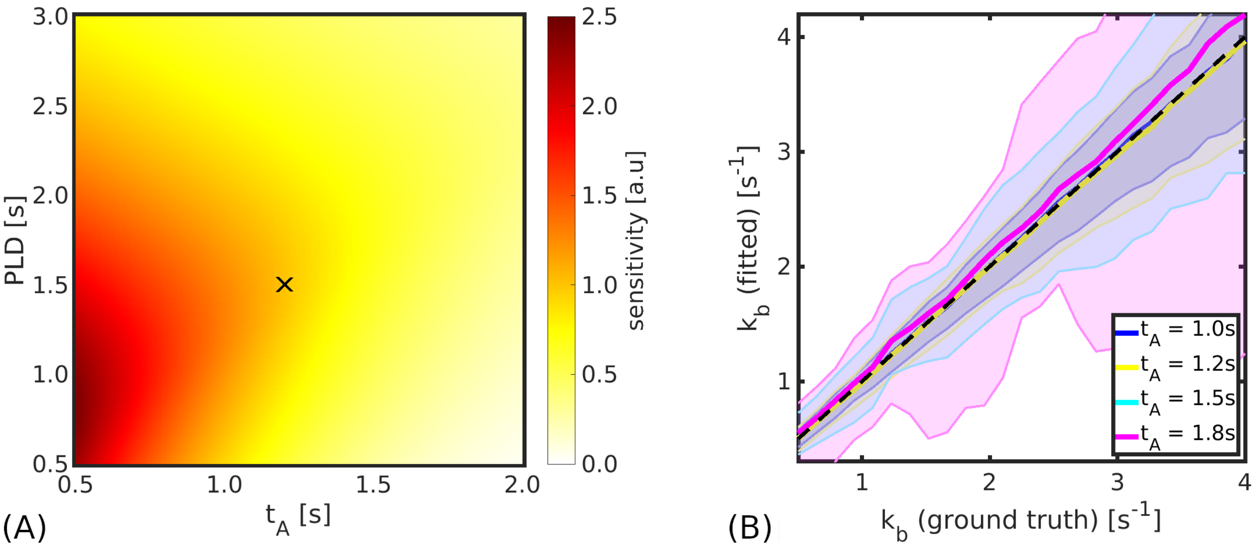

Figure S8. Effect of ATT variation.

(A). Sensitivity analysis (analogous to Figure 3A). To explore the combined effect of different ATT (t_A) and post label delay (PLD) times, the sensitivity functions (Equation 7) were computed for parameter combinations in the ranges $0.5\,\mathrm{s} \leq t_A \leq 2.0\,\mathrm{s}$ and $0.5\,\mathrm{s} \leq \mathrm{PLD} \leq 3.0\,\mathrm{s}$, with exchange rate $k_b = 2.65\,\mathrm{s}^{-1}$, extravascular $T_{1,e} = 1.5\,\mathrm{s}$ and optimum blood post-contrast $T_{1,b}^{post} = 0.8\,\mathrm{s}$. The colourbar shows the magnitude of the sensitivity functions, which were normalised using the value at $t_A = 1.2\,\mathrm{s}$ (i.e. corresponding to the parameter set used to normalise the sensitivity functions in Figure 3; indicated by the black cross). Other fixed parameters: $f = 60\,\mathrm{ml\,blood} / \mathrm{min} / 100\,\mathrm{ml\,tissue}$, $t_L = 2\,\mathrm{s}$, $\lambda = 0.9$ and $\alpha = 0.85$. **(B)**. Monte Carlo simulations (analogous to Figure 5A). The accuracy and precision of fitted k_b values over a range of ATT values were assessed using Monte Carlo simulations at a single noise level. Data were simulated for 25 k_b values between $0.5 - 4.0\,\mathrm{s}^{-1}$ and four ATT values of $t_A = 1.0, 1.2, 1.5, 1.8\,\mathrm{s}$. Other parameters: blood pre-contrast $T_{1,b}^{pre} = 1.65\,\mathrm{s}$, optimum blood post-contrast $T_{1,b}^{post} = 0.8\,\mathrm{s}$, $T_{1,e} = 1.5\,\mathrm{s}$, $f = 60\,\mathrm{ml\,blood} / \mathrm{min} / 100\,\mathrm{ml\,tissue}$, $t_L = 2\,\mathrm{s}$, $\lambda = 0.9$ and $\alpha = 0.85$. For each parameter combination, 2500 control and label signals were synthesised. Zero-mean Gaussian noise was added to the control and labelled data independently, giving a voxel-wise SNR of 30 in the control data. This voxel-level SNR was increased by $\sqrt{N}$ to simulate higher SNR at the cortical regional level, with $N = 500$ taken as the approximate number of voxels in a cortical region. Shaded regions indicate the IQR of fitted k_b values for each parameter combination; black dashed lines indicate ground truth values.

Supporting information S9: systematic errors in blood $T_{1,b}$ values (simulated data)

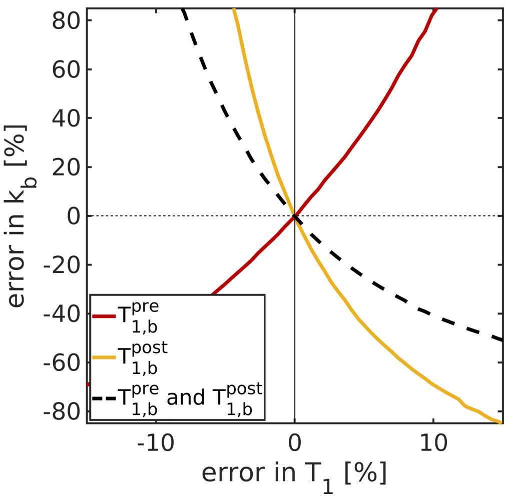

Figure S9. Effect of systematic blood $T_{1,b}$ errors (simulated data).

The error propagated into the exchange rate estimates, k_b, from systematic errors affecting blood pre-contrast $T_{1,b}^{pre}$ and blood post-contrast $T_{1,b}^{post}$ values equally (black dashed line); errors propagated into k_b from errors in $T_{1,b}^{pre}$ and $T_{1,b}^{post}$ separately are shown again for completeness (red and yellow lines). Ground truth parameter values: $k_b = 2.65\,\mathrm{s}^{-1}$, $f = 60\,\mathrm{ml\,blood\,/\,min\,/\,100\,ml\,tissue}$, $t_A = 1.2\,\mathrm{s}$, $T_{1,b}^{pre} = 1.65\,\mathrm{s}$, $T_{1,b}^{post} = 0.8\,\mathrm{s}$, $T_{1,e} = 1.5\,\mathrm{s}$, 5 pre-contrast PLDs between $0.9 - 2.1\,\mathrm{s}$, 1 post-contrast PLD $= 1.5\,\mathrm{s}$, $t_L = 2\,\mathrm{s}$, $\lambda = 0.9$ and $\alpha = 0.85$.

Supporting information S10: systematic errors in blood $T_{1,b}$ values (in vivo data)

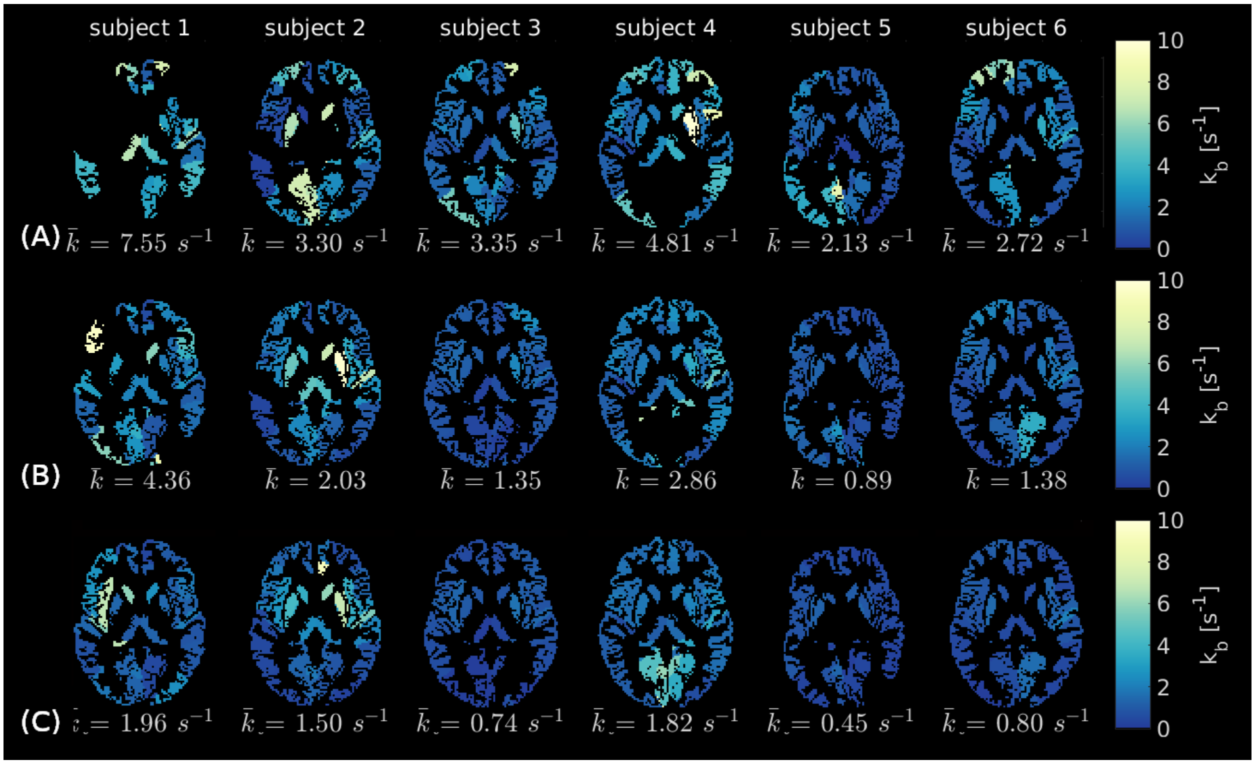

Figure S10. Effect of systematic blood $T_{1,b}$ errors (in vivo; PC2).
(A). Estimated regional exchange rate, k_b, obtained with blood pre-contrast $T_{1,b}^{pre}$ and blood post-contrast $T_{1,b}^{post}$ values adjusted 10 % lower than measured. **(B)**. Estimated k_b with $T_{1,b}^{pre}$ and $T_{1,b}^{post}$ as measured (i.e. as in Figure 7). **(C)**. Estimated k_b with $T_{1,b}^{pre}$ and $T_{1,b}^{post}$ adjusted 10 % higher than measured. All parameter values derived from the second post-contrast data set, PC2. In all maps, black voxels represent masked white matter and CSF, as well as extreme parameter fits ($k_b < 0\,\mathrm{s}^{-1}$ or $k_b > 10\,\mathrm{s}^{-1}$). Exchange rates averaged over the ROIs ($\bar{k}$) are displayed for each volunteer.
